# Ancient DNA of Guinea Pigs (*Cavia* spp.) Indicates a Probable New Center of Domestication and Pathways of Global Distribution

**DOI:** 10.1038/s41598-020-65784-6

**Published:** 2020-06-01

**Authors:** E. Lord, C. Collins, S. deFrance, M. J. LeFebvre, F. Pigière, P. Eeckhout, C. Erauw, S. M. Fitzpatrick, P. F. Healy, M. F. Martínez-Polanco, J. L. Garcia, E. Ramos Roca, M. Delgado, A. Sánchez Urriago, G. A. Peña Léon, J. M. Toyne, A. Dahlstedt, K. M. Moore, C. Laguer Diaz, C. Zori, E. Matisoo-Smith

**Affiliations:** 1 University of Otago, Dunedin, New Zealand; 2 Stockholm University, Stockholm, Sweden; 3 University of Florida, Gainesville, FL 32611 United States; 4 Florida Museum of Natural History, Gainesville, FL 32611 United States; 5 University College Dublin, Dublin, Ireland; 6 Université Libre de Bruxelles, Brussels, Belgium; 7 University of Oregon, OR, 97403 United States; 8 Trent University, Ontario, Canada; 9 Universitat Rovira i Virgili (URV), Tarragona, Spain; 10 Muséum National d’histoire Naturelle, Paris, France; 11 Institut Català de Paleoecologia Humana i Evolució Social, Tarragona, Spain; 12 Stetson University, DeLand, FL 32723 United States; 13 University of Los Andes, Bogotá, Colombia; 14 Consejo Nacional de Investigaciones Científicas y Técnicas, Buenos Aires, Argentina; 15 Facultad de Ciencias Naturales y Museo, La Plata, La Plata, Argentina; 16 School of Life Sciences and Human Phenome Institute Fudan University, Shanghai, China; 17 Instituto Colombiano de Antropología e Historia, Bogotá, Colombia; 18 National University of Colombia, Bogotá, Colombia; 19 University of Central Florida, Orlando, FL 32816-1361 United States; 20 Arizona State University, Tempe, AZ 85281 United States; 21 University of Pennsylvania, Philadelphia, PA 19104 United States; 22 Baylor University, Waco, TX 76798 United States

**Keywords:** Genomics, Archaeology

## Abstract

Guinea pigs (*Cavia* spp.) have a long association with humans. From as early as 10,000 years ago they were a wild food source. Later, domesticated *Cavia porcellus* were dispersed well beyond their native range through pre-Columbian exchange networks and, more recently, widely across the globe. Here we present 46 complete mitogenomes of archaeological guinea pigs from sites in Peru, Bolivia, Colombia, the Caribbean, Belgium and the United States to elucidate their evolutionary history, origins and paths of dispersal. Our results indicate an independent centre of domestication of *Cavia* in the eastern Colombian Highlands. We identify a Peruvian origin for the initial introduction of domesticated guinea pigs (*Cavia porcellus*) beyond South America into the Caribbean. We also demonstrate that Peru was the probable source of the earliest known guinea pigs transported, as part of the exotic pet trade, to both Europe and the southeastern United States. Finally, we identify a modern reintroduction of guinea pigs to Puerto Rico, where local inhabitants use them for food. This research demonstrates that the natural and cultural history of guinea pigs is more complex than previously known and has implications for other studies regarding regional to global-scale studies of mammal domestication, translocation, and distribution.

## Introduction

The use of ancient DNA (aDNA) in studies of animal domestication and subsequent translocation has radically improved our ability to identify spatially, temporally, and culturally variable processes of domestication and the diversity of social networks behind domestic species distribution (e.g.^1,2^). Increasingly, aDNA studies are revising previous assumptions of geographically conscripted animal domestication and dispersal events to reveal multiple centers, timings, and processes of domestication of the world’s most prominent domestic animals (e.g. pigs, chickens, cattle, dogs^3–6^). Because domestic animals are exemplar proxies for investigating past human migration and interaction, understanding long-term, diachronic patterns of when and where species domestication and translocation took place is critical to modelling past human use of and impacts on domestic animal genetic diversity within contexts of regional and global human interaction networks^7^.

In the Americas, animal domestication is documented in South and Central America where people domesticated llamas (*Lama glama*), alpacas (*Vicugña pacos*), guinea pigs (*Cavia porcellus*), Muscovy ducks (*Cairina moschata*), and turkeys (*Meleagris gallopavo*)^8,9^. The evolutionary history of guinea pig domestication and translocation beyond the Andean region warrants attention because of their long-term and diverse relationships with humans, including their ongoing economic importance in the Central Andes, their contemporary worldwide distribution as both pets and laboratory specimens, and their growing importance as a popular micro-livestock in other world areas (e.g. Africa).

For millennia, Central Andean peoples used guinea pigs (*Cavia* species) as a food source and for ritual and medicinal purposes^10^. The earliest archaeological remains of wild guinea pigs are radiocarbon dated to around 9,000 BC from sites in the eastern highlands of Colombia^11^. Early archaeological remains are also recorded from Jaywamachay, Peru (8500–8160 BC) and in northern Chile (from 8000 BC)^12,13^. Previous archaeological evidence from sites in the Central Andes indicates that guinea pigs were domesticated sometime between 6000 and 2000 BC^9,14^. Molecular analyses^15,16^ show that *Cavia tschudii* was the probable ancestor of the domestic *C. porcellus*. Spotorno *et al*.^16^ suggest that there were three phases of human interaction with guinea pigs: the initial domestication from *C. tschudii* to *C. porcellus*, which probably occurred in southern Peru/northern Chile, followed by two subsequent modern selection processes outside of South America resulting in the laboratory and pet breeds of Europe and improved breeds for the South American meat market.

Some researchers, however, suggest there were multiple locations of domestication of *Cavia* within South America and possibly the domestication of other *Cavia* species^14,15^. The oldest archaeological specimens (9000 BC) recovered from a cultural context are located in the Sabana de Bogotá, Colombia, which indicates early utilization of *Cavia* in this region. Initial analyses by Ijzereef^14^, based on morphological changes identified in *Cavia* skeletal remains (skulls and pelvises), suggest that after a long history of hunting wild guinea pigs, the Herrera period (~800 BC-800 AD) peoples independently domesticated Colombian guinea pigs around 500 BC. However, more recent osteological research on modern and archaeological specimens of the three species present in Colombia today - *C. anolaimae* in the highlands and savanna of Bogotá; *C. (aperea) guianae* in the northeast; and *C. porcellus* in the south - suggests that domestic guinea pigs may have been present in Sabana de Bogotá as early as the Late Glacial and early Holocene (11000–9000 BC)^17^.

Based on archaeological, bioarchaeological and isotopic evidence Delgado^18–20^ suggests that important changes in the relationship between guinea pigs and humans occurred in the Bogotá Highlands during the late Holocene (3180–500 BC). This is linked to the probable arrival of new people to the region from the lowlands, likely introducing agriculture^21^. Isotopic evidence of a mixed C3/C4 diet indicates feeding of *C. porcellu*s at a late Muisca site of Tibanica (Cal AD 1005–1380) in the Sabana de Bogotá^18^. Human feeding might indicate a possible commensal pathway in the process of domestication; however, feeding either wild or feral guinea pigs might also indicate tending (see^22^). Domesticated guinea pigs may have coexisted with wild or feral varieties in Colombia^17,23,24^, with remains of both *C. porcellus* and *C. anolaimae* described (morphologically) from the sites of Checua and Aguazuque^21^. Guinea pigs included in burials at both the Late Herrera Period (AD 900) and the Late Muisca Period contexts (AD 1250–1600) at the El Venado site indicate a symbolic, mortuary relationship had developed between humans and guinea pigs in conjunction with the economic role of these animals. The presence of multiple species of *Cavia* remains in Colombia in both economic and religious contexts through time indicates a complex evolutionary history and a potential independent domestication of *Cavia* in northern Colombia.

Within the Central Andes, following their domestication, archaeological evidence shows that people dispersed *C. porcellus* through much of South America, including Chile, Argentina, western Brazil, Bolivia, and Ecuador (the latter likely via the well-documented maritime trade in spiny oysters (*Spondylus* sp.)^25,26^). By AD 600, *C. porcellus* remains are present on some Caribbean islands, where they were likely used as a supplementary food source to the primarily horticultural and marine-based economy^27,28^. Current archaeological evidence suggests that people introduced guinea pigs first to Puerto Rico and later to the rest of the Greater and the Lesser Antilles^27,29^. The majority of pre-Columbian Caribbean guinea pig remains are from sites on Puerto Rico, which includes the largest known assemblage of remains from the Finca Valencia site^29^.

An initial aDNA study investigating the origin and dispersal of ancient guinea pigs in the Caribbean, which focused on the small hypervariable portion of the mitochondrial DNA (mtDNA), suggested the ancient Caribbean samples were most closely related to modern guinea pigs purchased from markets in Colombia, implying a trade link or other type of interaction between the two areas^30^. This explanation was considered parsimonious in light of known interaction networks between the Caribbean islands, the Isthmus of Panama, and northern South America^31^. In subsequent analyses of complete mitogenome diversity of three ancient Caribbean samples, Lord *et al*.^32^ identified two probable guinea pig introductions to the Caribbean during the Late Ceramic Age (ca. AD 500–1500), although the origin of these migrations could not be determined due to a lack of comparative guinea pig aDNA data generated from South American sites.

Soon after early European contact with South America and the Caribbean in the late 15^th^ century, people transported guinea pigs to Europe where they were initially sought-after pets for the European upper class, only later becoming accessible to the other classes. Beginning in the 18^th^ century, medical researchers of the time used guinea pigs as laboratory animals for study^33,34^. The oldest remains of guinea pigs in Europe date to the second half of the 16^th^ century and come from Mons, Belgium (AD 1550–1640)^35^ and Hill Hall Manor, England (AD 1574–1575)^36^. Spotorno *et al*.^16^ suggest that European guinea pigs originated in Peru. People also transported guinea pigs to North America, where the earliest evidence is found in Charleston, South Carolina, dating to the early 19^th^ Century^37^.

**Figure 1:**
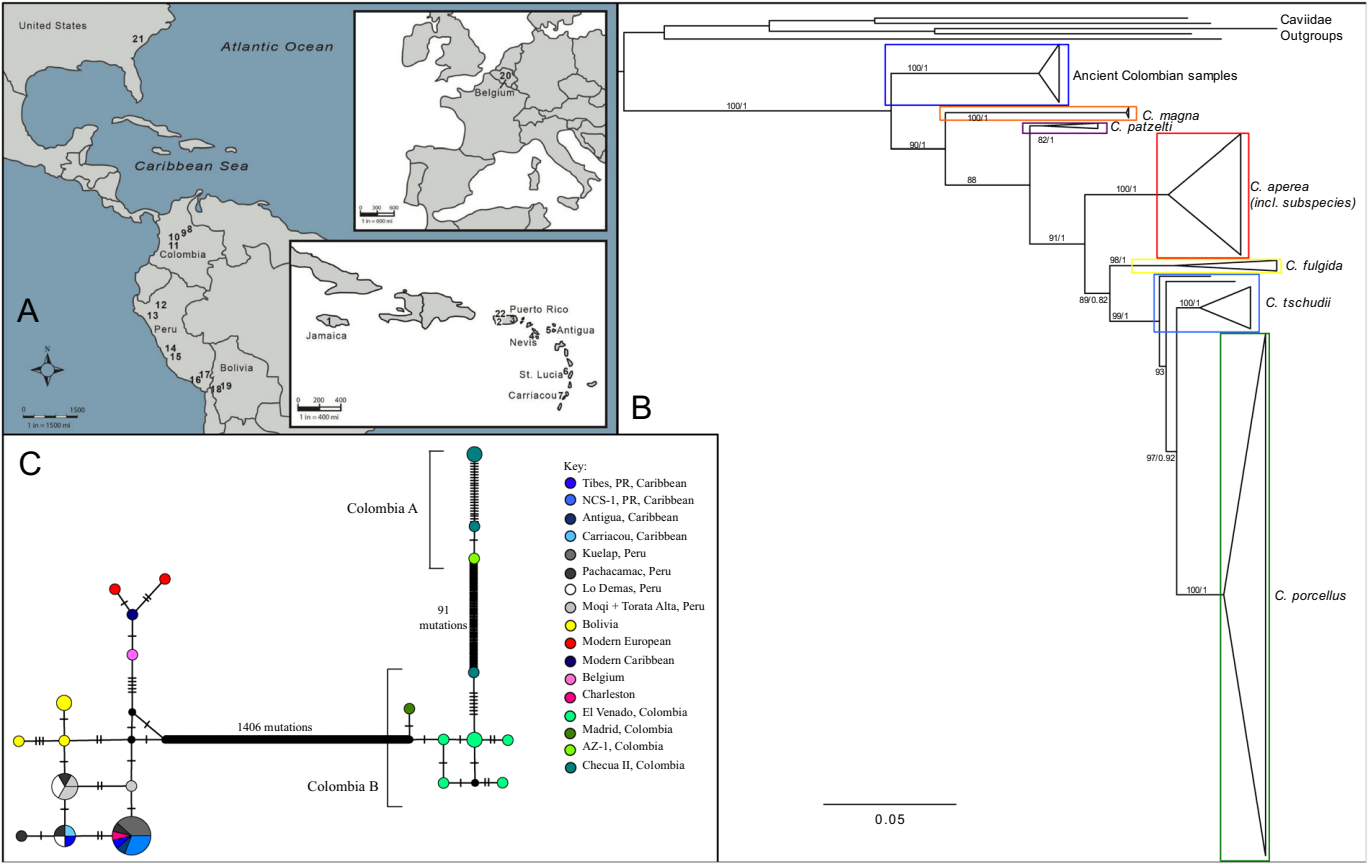
**A**) Map of archaeological sites included in the study. 1. Green Castle, Jamaica, 2. Tibes, Puerto Rico, 3. Finca Valencia (NCS-1), Puerto Rico, 4. Coconut Walk, St Kitts and Nevis, 5. Coconut Hall, Antigua, 6. Giraudy, St Lucia, 7. Grand Bay, Carriacou, 8. El Venado, Colombia, 9. Checua II, Colombia, 10. Madrid Site, Colombia, 11. Aguazuque, Colombia, 12. Kuelap, Peru, 13. Zana, Peru, 14. Pachacamac, Peru, 15. Lo Demas, Peru, 16. Torata Alta, Peru, 17. Moqi, Peru, 18. Kala Uyuni, Bolivia, 19 Llusco Structure, Chiripa, Bolivia, 20. Mons, Brussels, Belgium, 21. Charleston, South Carolina, United States, 22. San Sebastian, Puerto Rico. Figure was created using Adobe Illustrator CS5 6.0 with an open access base map from University of Florida Map Library. (**B**) Cytochrome B Phylogeny of Caviidae species (sequences available on Genbank). *Cavia* spp. are boxed. (**C**) Median Joining network of all complete mitogenomes and the reference sequence (Modern European). Samples are coloured by site. The size of the circle represents the number of samples sharing that haplotype. Black circles represent intermediate haplotypes.

In order to better understand guinea pig domestication and dispersal, we attempted to sequence complete mitochondrial genomes (mitogenomes) from ancient guinea pig specimens morphologically identified as *Cavia porcellus* from 19 pre-Columbian archaeological sites in Colombia, Peru, Bolivia and the Caribbean Antilles, along with two historic samples of guinea pigs in Europe (Mons, Belgium) and North America (Charleston, South Carolina), and one modern sample from Puerto Rico (Fig. 1A) (SI Table S1). This collection of samples allows us to elucidate the evolutionary history of domestic guinea pigs and to identify the distribution routes of *Cavia porcellus* outside of South America into the Caribbean Antilles and beyond.

## Results

### DNA preservation, sequence authenticity and contamination

Of 66 archaeological samples processed, 59 produced DNA libraries adequate for mitogenome sequencing. Post sequencing, 42 samples provided over 90% coverage of the mitogenome and were retained for further analysis, giving an overall success rate of 64% (SI Table S2). Samples had an average read depth of 654×, ranging from 4.7× to 3297× (SI Tables S3 and S4). All samples showed damage patterns and fragment lengths characteristic of aDNA with no indication of contamination from lab reagents or other sources (SI Table S3).

Poor DNA preservation and lower success rates were generally seen in samples from coastal Caribbean and Peruvian sites. Four sites, in Jamaica, Nevis and St Lucia in the Caribbean, and Carrizales in Peru, failed to yield any usable data (SI Table S4). This is likely due to the coastal environment not being conducive to good DNA preservation, a result reported in other aDNA studies for the Caribbean, as well as from other coastal, tropical environments^38–44^. In addition, only one of three samples from Aguazuque I (1885–1815 BC), in the eastern Colombian Highlands, produced a complete mitogenome, likely due to a combination of age and environmental factors. Four of the five samples processed from the older site of Checua II (5940–5670 BC), also in the eastern Colombian Highlands, however, produced complete mitogenomes, as did all six of the samples from El Venado (AD 800–1600) as well as one sample from the Madrid site (200–100 BC). The cultural affiliations and additional information regarding the context of all archaeological samples used in our phylogenetic analyses are provided in Table S5.

### Phylogenetic identity of Colombian samples

When we initially attempted to map all of our sequences to the *C. porcellus* reference mitogenome (Genbank ID: NC_000884.1), we found that none of the 12 Colombian samples mapped efficiently and all showed an identical pattern of alignment/coverage (SI Fig. S1). This pattern led us to suspect that they may not be *C. porcellus*. Thus, a de novo assembly was carried out using one high coverage Colombian sample (MS10677, from Checua II) to produce a consensus Colombian reference sequence, to which all other Colombian samples were subsequently mapped (SI Fig. S2). This produced a much higher mapping success from all Colombian samples.

Given the lack of comparative complete mitogenomes for *Cavia* species, we compared the Cytochrome B region from our Colombian samples with all Caviidae data available in Genbank (SI Table S6). The resulting Maximum Likelihood consensus tree (Fig. 1B) suggests that the ancient Colombian archaeological remains appear to represent a distinct *Cavia* species; none of the 12 sequenced Colombian samples fell within the *C. porcellus* clade.

### Relationships identified using complete mitogenomes

Once all Colombian samples were realigned to the consensus Colombian reference sequence, a Median Joining (MJ) network (Fig. 1C) was constructed using all of the complete mitogenomes generated in this study, as well as the *C. porcellus* reference mitogenome (Genbank: NC_000884.1). The *C. porcellus* and ancient Colombian samples are separated by 1406 mutations, as seen in the CytB tree (Fig. 1B), suggesting that they are from two distinct species.

Within the Colombian samples, two distinct groups are identified, with the two clades separated by 91 mutations. The separation of these samples, all identified as belonging to the same species based on analysis of the Cyt B region, appear to correlate temporally. The first, labelled Colombia A (Fig. 1C), contains samples from the older contexts, Checua II (5940–5760 BC) and Aguazuque (1885–1815 BC). The second group, Colombia B, consists of samples from the younger contexts, Madrid (200–100 BC) and El Venado (AD 800–1600), except for a single sample from Checua II.

Within the *C. porcellus* samples, we see three distinct groups: Caribbean and Peruvian samples, the Bolivian samples, and the historic/modern European samples. Our analyses of eight samples from four sites in the Caribbean identify two haplotypes separated by two mutations. One haplotype contains samples from northern Peru (Kuelap), a single sample from coastal Peru (Pachacamac), Puerto Rican samples from both Tibes (Tibes A) and Finca Valencia and a sample from Antigua. The second haplotype was found in samples from Tibes (Tibes B) and Carriacou, as well as samples from southern and coastal Peru (Pachacamac, Lo Demás, Moqi and Torata Alta).

The two historic samples had sequences that clearly place them within *C. porcellus*. The South Carolina sample shared a haplotype with the ancient Peruvian and Puerto Rican (Kuelap, Tibes A) samples, whereas the Belgian sample clustered with the modern samples (the reference sequence NC_000894.1, and the modern sample from Lord *et al*.^32^), and the modern Caribbean sample.

## Discussion

### Cavia taxonomy and a unique process of domestication in Colombia

Our analyses of the Cyt B data show that all 12 of our ancient Colombian samples fall into a distinct clade that is basal to all other members of the *Cavia* genus (Fig. 1B, SI Fig. S4). When we compare the analysis of the complete mitogenomes (Fig. 1C), we see that the number of mutations between the ancient Colombian samples and the *C. porcellus* samples (n = 1406) is similar to the level of variation reported between *Rattus* spp., such as *R. norvegicus* and *R. exulans* differing by 1577 mutations across the mitogenome^45^. We therefore suggest that the ancient Colombian samples we sequenced fall within the *Cavia* genus, but do not belong to *C. porcellus*, or other *Cavia* species for which there are mitochondrial data available. Interestingly, the ancient Colombian samples are genetically distinct from previously published modern Colombian (*C. porcellus*) sequences (SI Table S6), although these were sourced from southern Colombian markets, as opposed to the highlands of Bogotá where the ancient samples were located. These data suggest the utilization of two different species of guinea pig in Colombia through time: a native *Cavia* species, identified through the analyses of the archaeological remains studied here, and a secondary, more recent introduction of *C. porcellus* to coastal Colombia for the meat market.

All of the ancient Colombian samples in this study were previously morphologically identified as *C. porcellus*, however, as both *C. porcellus* and *C. anolaimae* have been described from the same occupation layers at Checua and Aguazuque, we tentatively suggest that all of the Colombian samples sequenced here represent *C. anolaimae*. First described by Allen in 1916^46^, the taxonomic position of *C. anolaimae* has been debated, most recently described as a subspecies (*C. aperea anolaimae*) or a synonym for the domestic *C. porcellus*^15^. Morphological and molecular evidence generally demonstrate it is a distinct species^46–48^, with the exception of one study^15^. Unfortunately, there is only a single Cytochrome B sequence available for *C. “aperea” anolaimae* in public databases (GU136758 in SI Fig. S4). The identification of this sample appears to be problematic based on the analyses reported here and by that of Dunnum and Salazar-Bravo^15^, as it falls clearly within the *C. aperea guianae* clade (SI Fig. S4). We suggest that this sample is either mislabeled or misidentified, meaning there are no reliable Cytochrome B data for *C. anolaimae*. Obtaining a reliable reference sequence for this species is crucial for a definitive species identification of our Colombian samples, though we expect that they may represent *C. anolaimae*.

Our mitochondrial genome analyses (Fig. 1C) show the presence of two distinct groups/clades within the ancient Colombian guinea pigs, which are separated temporally by more than 1000 years. Campos and Ruiz-Garcia^48^ also found a distinction between two modern populations of *C. anolaimae*, which the authors attributed to geographical isolation. However, given the antiquity, the use of guinea pigs as food, and their inclusion in burials at some archaeological sites in the highlands of Colombia, we suggest the genetic change over time represented by our data may be indicative of an independent domestication of a native *Cavia*, tentatively identified as *C. anolaimae*, from a diverse wild population. It is plausible that sustained interaction with humans in Colombia resulted in a process of commensal domestication as defined by Zeder^22^, possibly in association with agriculturalists moving into the highlands^21^, or even in the Late Glacial as suggested by Pinto *et al*.^17^. This supports previous suggestions of an independent *Cavia* domestication in Colombia based on morphological analyses^14,17–20^, although specific the timing of this process remains unclear.

The significance of the two distinct groups of Colombian guinea pigs warrants further study of this possible process of domestication, independent of that which led to *C. porcellus* in the Central Andes. Alternatively, our samples may represent either a wild *Cavia* species or an extinct species that did not contribute genetically to the modern populations, even though this species is present in archaeological sites up until AD 1600 indicating that it was present in the highlands until after European colonization began. Furthermore, we suggest that the modern Colombian guinea pigs (from markets) are not related to the archaeological population from the highlands, but are descended from the historic and modern redistribution of varieties bred intentionally for the meat market. Further sequencing and morphological study of ancient and modern *Cavia* species from the highlands of Colombia will clarify the origins and evolutionary history of Colombian guinea pigs, and confirm whether this species represents an independent process of domestication of *Cavia*.

### The origins of guinea pig translocation to the Caribbean Antilles

Previous aDNA studies^30,32^ suggested a possible Colombian origin for Caribbean guinea pigs. Our results, as shown in the haplotype network (Fig. 1C), indicate that all Caribbean samples share haplotypes with samples from Peru. Thus, we suggest a probable Peruvian origin of the guinea pigs that people translocated to the Caribbean Antilles. This is significant, as no direct connections to Peru have been established empirically through previous archaeological studies. Although similarities in pottery style and raptoral bird iconography are noted between sites on Puerto Rico, Vieques, and in northwestern South America, including the eastern foothills of the Andes^49,50^, the geographic specificity of links between the Caribbean Antilles and South American Andes have remained largely speculative (e.g.^51^). Thus, this study significantly extends our knowledge of the Late Ceramic Age Caribbean-South American interaction spheres.

The ancient Caribbean guinea pig samples are most closely related to samples from Kuelap (AD 1100–1535) in the northern highlands of Peru, as well as to samples from the Peruvian coastal sites of Pachacamac (AD 600–1000 and AD 1470–1572) and Lo Demas (AD 1480–1540). Both Kuelap and Pachacamac were large sites/polities in late pre-Columbian Peru. The site of Kuelap was first inhabited as a religious center from AD 500, although it is dominated by monumental architecture of the Chachapoyas culture (AD 900–1470), and later the Inca (AD 1470–1535). The site of Pachacamac (AD 600–1500) was also associated with monumental architecture, though has successive Lima, Wari, Ychsma, Inca and early Colonial occupations^52^. This site likely was a ‘pilgrimage’ center for those travelling throughout Peru, such as from Lo Demas and beyond, although no Caribbean style artifacts have been recorded there. The identification of haplogroups shared between these sites in Peru and sites in the Caribbean Antilles, suggests that interaction between these groups and their wider community extended more broadly than previously documented.

### Number of translocations and possible routes to the Caribbean Antilles

**Figure 2:**
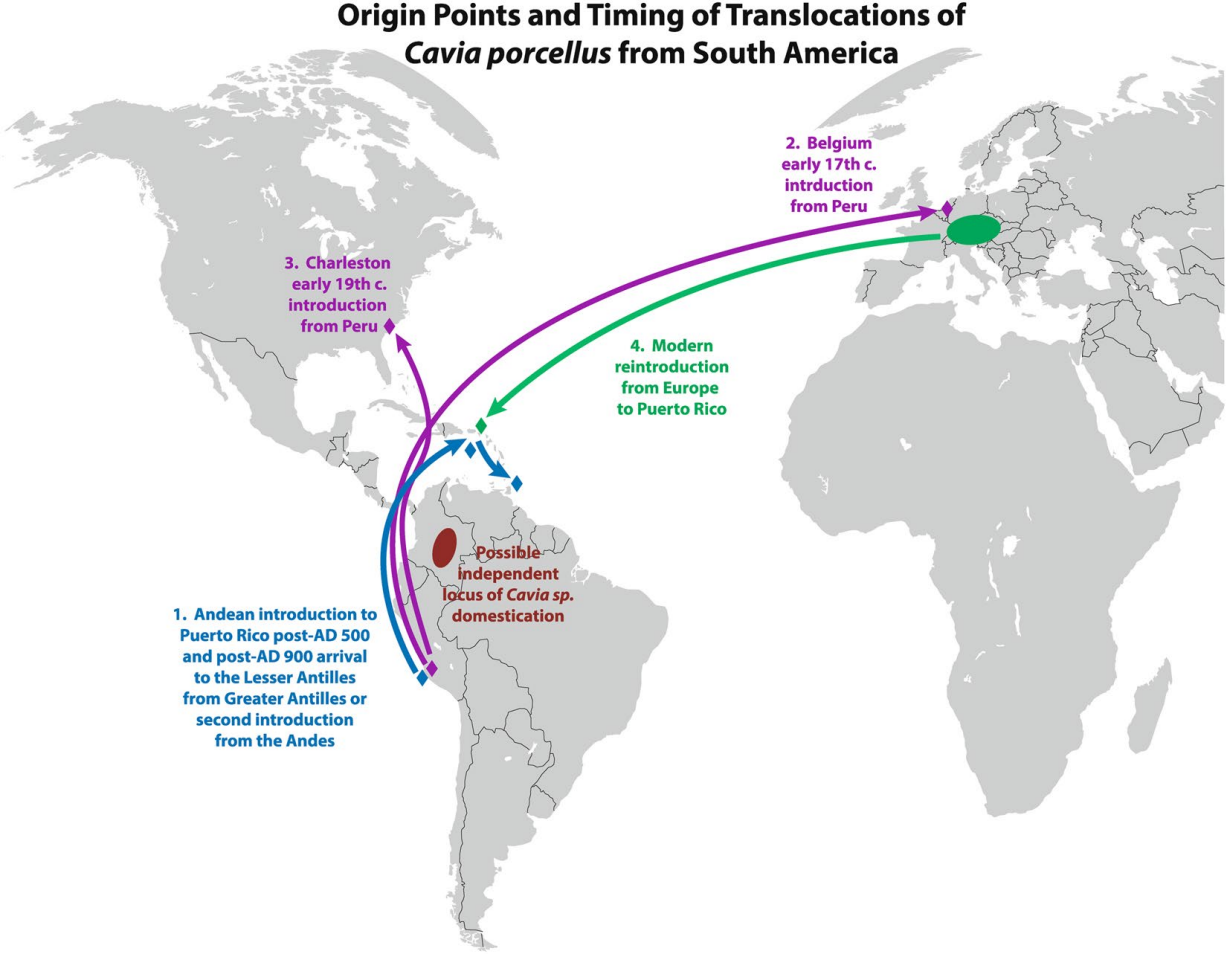
Origin points and timing of translocations of *Cavia porcellus* from the Central Andean region to the Pre-Colombian Caribbean, early modern Europe, colonial North America, and modern reintroduction from Europe to Puerto Rico. The figure also shows the region of a probable new center of Cavia domestication in Colombia. The routes depicted are purely hypothetical; people may have translocating guinea pigs using routes other than those depicted. Figure was created using Adobe Illustrator CS5 6.0 with an open access base map from University of Florida Map Library.

Previous analyses by Lord *et al*.^32^ identified two haplotypes in the three complete mitogenomes of ancient guinea pigs from three sites in the Caribbean, which also was supported by the increased sample size reported here. We suggest that the initial introduction likely occurred from a guinea pig population originally sourced from northern Peru that reached Puerto Rico around AD 600 (Fig. 2). Significantly, the Puerto Rican samples from both Tibes A and Finca Valencia share a single haplotype, suggesting an initial migration of a non-genetically diverse population. From this founding population, we suggest that guinea pigs were then dispersed to Antigua, in the northern Lesser Antilles after AD 1000, in line with cultural interaction during this time period^53–55^. The second Caribbean haplotype, found at Tibes B (Puerto Rico) and Carriacou, potentially represents a distinct translocation, either directly or indirectly from an ancestral population in coastal Peru, possibly around the site of Pachacamac. This scenario for a coastal origin is also supported by the presence of domesticated guinea pigs in coastal Ecuadorian sites and evidence for long-distance maritime trade of spiny oyster^25,26^. The possibility of multiple introductions of guinea pigs to the Caribbean further supports archaeological and isotopic evidence that the Late Ceramic Age cultures of the Caribbean, called Ostionoid (ca. AD 500-European contact ca. AD 1492), were highly mobile and had large, ongoing interaction networks reaching across the Caribbean to both Central America and northern South America (19, 44–46, 54).

The exact route of the initial or subsequent introduction(s) of guinea pigs through northwestern South America and into the Caribbean Antilles is difficult to determine. Translocation may have been the result of direct or indirect down-the-line trade networks via either, or a combination of: a northwestern coastal route, a Pacific maritime route and an eastern Andean overland route (given the genetic similarity to guinea pigs from the montane site of Kuelap). However, few archaeological specimens of domestic guinea pigs exist in northern South America, which makes potential translocation routes through this area difficult to discern. A single specimen has been identified from the inland site of Turen in northern Venezuela^56^ and eight individuals (morphologically identified as *C. aperea*) are reported from the Palmasola site near the northwest coast of Venezuela^57^ but these specimens could not be obtained for this study. To date, no known ancient guinea pig remains have been recovered from the coastal regions of Colombia. We observed poor aDNA preservation from samples recovered from coastal localities even when the archaeological specimens appeared to be well-preserved (SI Section 1.1, Table S4). Thus, it may be that remains of guinea pigs brought along either the western and northern coasts or the northeastern interior of South America just do not survive in the archaeological record of these regions. If taphonomic and preservation factors cannot account for the lack of archaeological guinea pig remains in coastal Colombia and Venezuela, it is possible that the movement of guinea pigs was rapid, thus leaving no archaeological signature.

From northern South America, guinea pigs may have been introduced to Puerto Rico via *guanín* trade with the Isthmo-Colombian region (as described in^50,58^) or the trade of Guatemalan-sourced jadeitite/jadeite artifacts from Central America to the Caribbean^59^. This could offer an alternative route of introduction of guinea pigs to the Caribbean and explain the second haplotype in Puerto Rico (Tibes B), which was then dispersed to Carriacou in the southern Lesser Antilles. This introduction possibly occurred around AD 900, which correlates with the development of increasingly hierarchical societies in Puerto Rico^60^. Thus, almost simultaneously, guinea pigs could have then been transported down to the Lesser Antilles through sustained inter-island trade and interactions.

It is also possible, however, that only a single introduction of guinea pigs to the Caribbean occurred, via the routes described above, with later dispersal within the Antilles. This hypothesis would support a previous proposition by Wing^29^ that the significant quantity of guinea pig bones from Finca Valencia on the north coast of Puerto Rico may indicate that Ceramic Age colonists established a founding population of guinea pigs on Puerto Rico and then translocated the animals to other islands. Additional analyses of DNA recovered from directly dated ancient Caribbean samples may be able to discern between the multiple and single introduction hypotheses and allow for better interpretation of the timing and route(s) of the introductions.

### Modern reintroduction to the Caribbean

To date, no archaeological remains of guinea pigs have been identified or verified from well-excavated and well-analyzed early Spanish colonial-associated Caribbean sites^27,61,62^. Our analysis of the mitogenome of a modern specimen from San Sebastían, Puerto Rico indicates that it is not related to any of the archaeological specimens in the Caribbean. Instead, it grouped within the modern European samples, suggesting a recent reintroduction of guinea pigs to Puerto Rico occurred from Europe or, perhaps more likely, from the markets of southern Colombia or Peru (Fig. 2). Further complete mitogenome analyses of modern samples from South America will allow for the origins of the modern translocation to be elucidated, as currently only Cytochrome B data are available.

### Historic translocations and interactions with Europe and North America

The historic samples from Mons, Belgium and Charleston, South Carolina appear to have origins in the Central Andes (Fig. 2). The Belgian sample falls within the modern European group; however, this group is related most closely to samples from either Bolivia or Peru. We suggest that during the Spanish colonial period, guinea pigs from the Andean region of Bolivia/southern Peru were transported to Europe. It is also possible that guinea pigs came to Europe from northern Chile, although samples from this area have not yet been analysed. In order to clarify the precise origins of European guinea pigs and the timing of their translocation, more analysis of samples from historic era contexts in South America and Europe are needed.

The Charleston, South Carolina guinea pig, dating to AD 1820, represents the earliest evidence of guinea pigs in North America. This sample shares a haplotype with samples from Kuelap in northern Peru, and with specimens from Puerto Rico (e.g. Finca Valencia and the Tibes A specimen). As previously mentioned, there is no archaeological evidence for guinea pigs in Puerto Rico or elsewhere in the Caribbean during the colonial period, thus the Charleston specimen probably originated from Peru. Furthermore, the Charleston guinea pig was discarded in a privy, along with the remains of an Amazonian parrot^63^, suggesting that both animals were acquired as South American ‘curiosities’ (see SI information on Heyward-Washington House).

## Conclusions

Here we use mitogenome sequence data obtained from archaeological, historical, and contemporary guinea pigs to identify diachronic human networks of interaction spanning several millennia. Ancient mitogenomes obtained from *Cavia* remains recovered from archaeological sites in the Colombian Highlands were clearly genetically distinct from all other populations studied, suggesting that all of the ancient Colombian samples we analysed belong to a different *Cavia* species. We tentatively identify this species as *C. anolaimae*, based on previous literature describing the biogeography of this species. Unfortunately, no comparative mitogenome data for *C. anolaimae* exists in any public databases, so we cannot confirm this tentative species identification. Our result, however, supports previous suggestions that an independent guinea pig domestication may have occurred in the Colombian Highlands. Further genetic sampling as well as morphometric analyses of modern and archaeological guinea pig specimens will be necessary to broaden our understanding of the domestication of guinea pigs, and aid in identifying processes and biological outcomes of guinea pig domestication.

Our results also demonstrate that beginning around AD 600, people transported guinea pigs (*C. porcellus*) beyond the South American mainland, introducing them to the Caribbean Antilles from a Peruvian-derived population, possibly two times, through existing social networks of interaction and trade routes. In the absence of archaeological evidence for guinea pigs along coastal Central America, coastal Colombia, or elsewhere in northern coastal South America, this connection to modern Peru is the first empirical evidence demonstrating interactions between groups in the Caribbean Antilles and this region of western South America. We also identify a Central Andean origin of the earliest historic-era introductions of domestic guinea pigs into Europe and North America. Lastly, we identify a modern reintroduction of guinea pigs to the island of Puerto Rico, from Europe or, perhaps more likely, from the modern market breeds of South America.

This study adds to the growing body of evidence suggesting highly diverse geographical, temporal and cultural histories of mammal domestication and subsequent distribution. Through this analysis of ancient guinea pig mitogenomes, the history of such variability within the context of human social interactions over thousands of years and across three continents provides a critical historical perspective of the genetic diversity of one of the world’s oldest and enduring domestic animals.

## Materials and Methods

A total of 66 archaeological guinea pig samples from 21 sites were obtained from four archaeological sites in Colombia (n = 15), six sites in Peru (n = 32), two sites in Bolivia (n = 4), seven sites from the Caribbean (n = 13), two historic sites from Europe (n = 1) and North America (n = 1), and one modern market in Puerto Rico (Fig. 1A, Table S1). In addition, the modern European sample analysed in Lord *et al*.^32^ was included in downstream analyses. Specific details of archaeological sites and contexts of the analyzed specimens are presented in the Supplementary Information Section 1.1.

All archaeological samples were processed in a purpose-built ancient DNA facility at the University of Otago^64^. DNA extraction, library preparation, hybridization capture and sequencing methods, and description of bioinformatic processing of sequences are described in the electronic Supplemental Information Section 1.2.

## Supplementary information

Supplementary Information.

## References

[1] Orlando L (2020). Ancient Genomes Reveal Unexpected Horse Domestication and Management Dynamics. BioEssays.

[2] Irving-Pease Evan K., Ryan Hannah, Jamieson Alexandra, Dimopoulos Evangelos A., Larson Greger, Frantz Laurent A. F. (2018). Paleogenomics of Animal Domestication. Population Genomics.

[3] Verdugo MP (2019). Ancient cattle genomics, origins, and rapid turnover in the Fertile Crescent. Science (80-.)..

[4] Frantz LAF (2016). Genomic and archaeological evidence suggest a dual origin of domestic dogs. Science (80-.)..

[5] Frantz LAF (2019). Ancient pigs reveal a near-complete genomic turnover following their introduction to Europe. Proc. Natl. Acad. Sci. USA.

[6] Storey AA (2012). Investigating the Global Dispersal of Chickens in Prehistory Using Ancient Mitochondrial DNA Signatures. PLoS One.

[7] Bruford MW, Bradley DG, Luikart G (2003). DNA markers reveal the complexity of livestock domestication. Nature Reviews Genetics.

[8] Thornton EK (2012). Earliest Mexican Turkeys (Meleagris gallopavo) in the Maya Region: Implications for Pre-Hispanic Animal Trade and the Timing of Turkey Domestication. PLoS One.

[9] Wing, E. S. Animal domestication in the Andes. in Advances in Andean archaeology (ed. Browman, D. L.) 167–188 (Mouton Publishers, 1978).

[10] Sandweiss DH, Wing ES (1997). Ritual Rodents: The Guinea Pigs of Chincha, Peru. J. F. Archaeol..

[11] Correal, G. & der Hammen, T. Investigaciones arqueológicas en los abrigos rocosos del Tequendama: 11.000 años de prehistoria en la Sabana de Bogotá. Bogotá (Biblioteca Banco Popular, 1977).

[12] Stahl Peter W. (2008). Animal Domestication in South America. The Handbook of South American Archaeology.

[13] Hesse B (1984). Archaic exploitation of small mammals and birds in Northern Chile. Estud. Atacameños.

[14] Ijzereef G (1978). Faunal remains from the El Abra rock shelters (Colombia). Palaeogeogr. Palaeoclimatol. Palaeoecol..

[15] Dunnum JL, Salazar-Bravo J (2010). Molecular systematics, taxonomy and biogeography of the genus Cavia (Rodentia: Caviidae). J. Zool. Syst. Evol. Res..

[16] Spotorno AE (2006). Ancient and modern steps during the domestication of guinea pigs (Cavia porcellus L.). J. Zool..

[17] Pinto, M., Zúñiga, H. & Torres, O. Estudio Sistemático Del Género Cavia Pallas, 1766 (Rodentia:Caviidae) En Colombia. (Academia de Ciencias Exactas, Físicas y Naturales, 2002).

[18] Delgado ME (2018). Stable isotope evidence for dietary and cultural change over the Holocene at the Sabana de Bogotá region, Northern South America. Archaeol. Anthropol. Sci..

[19] Delgado ME (2017). Holocene population history of the Sabana de Bogotá region, Northern South America: An assessment of the craniofacial shape variation. Am. J. Phys. Anthropol..

[20] Delgado ME (2015). Mid and late Holocene populations changes at the Sabana de Bogotá (Northern South America) inferred from skeletal morphology and radiocarbon chronology. Quat. Int..

[21] Martínez-Polanco MF (2019). Beyond white-tailed deer hunting in Aguazuque: Archaeofaunal data from an archaic site at Sabana de Bogotá, Colombia. Int. J. Osteoarchaeol..

[22] Zeder MA (2015). Core questions in domestication research. Proc. Natl. Acad. Sci. USA.

[23] Martínez-Polanco María Fernanda (2016). El Cuy (CaviaSp.), Un Recurso Alimenticio Clave en Aguazuque, Un Sitio Arqueológico de la Sabana de Bogotá, Colombia. Latin American Antiquity.

[24] Garcia, J. L. The Foods and Crops of the Muisca: A Dietary Reconstruction of the Intermediate Chiefdoms of Bogotá (Bacatá) and Tunja (Hunza), Colombia. (Citeseer, 2012).

[25] Stahl PW (2003). Pre-Columbian Andean animal domesticates at the edge of empire. World Archaeol..

[26] Stahl PW, Norton P (1987). Precolumbian Animal Domesticates from Salango, Ecuador. Am. Antiq..

[27] LeFebvre MJ, deFrance SD (2014). Guinea Pigs in the Pre-Columbian West Indies. J. Isl. Coast. Archaeol..

[28] LeFebvre, M. J. & deFrance, S. D. Animal management and domestication in the realm of Ceramic Age farming. in The Archaeology of Caribbean and Circum-Caribbean Farmers (600 BC- AD 1500) (ed. Reid, B. A.) (Routledge, 2018).

[29] Wing, E. Guinea pig (Cavia porcellus) remains from Finca Valencia (NCS-1), Northwest Puerto Rico, contribution to Phase II Zooarchaeology at the Finca Valencia (NCS-1) and NCS-4 Site, Northwest Puerto Rico. Final Phase II Archaeological Evaluation for the Finca Valencia Site North Coast Superaqueduct Project Municipality of Arecibo, Puerto Rico. (2000).

[30] Kimura BK (2016). Origin of pre-Columbian guinea pigs from Caribbean archeological sites revealed through genetic analysis. J. Archaeol. Sci. Reports.

[31] Pagán-Jiménez JR, Rodríguez-Ramos R, Reid BA, van den Bel M, Hofman CL (2015). Early dispersals of maize and other food plants into the Southern Caribbean and Northeastern South America. Quat. Sci. Rev..

[32] Lord E, Collins C, deFrance SD, LeFebvre MJ, Matisoo-Smith E (2018). Complete mitogenomes of ancient Caribbean Guinea pigs (Cavia porcellus). J. Archaeol. Sci. Reports.

[33] Endersby, J. A guinea pig’s history of biology: the plants and animals who taught us the facts of life. (Random House, 2012).

[34] Wagner, J. E. Chapter 1 - Introduction and Taxonomy. in The Biology of the Guinea Pig (eds. Wagner, J. E. & Manning, P. J.) 1–4 (Academic Press, 1976).

[35] Pigière F, Van Neer W, Ansieau C, Denis M (2012). New archaeozoological evidence for the introduction of the guinea pig to Europe. J. Archaeol. Sci..

[36] Hamilton-Dyer, S. Animal Bones. in Hill Hall: A Singular House Devised by a Tudor Intellectual. (eds. Dury, P. & Simpson, R.) 345–351 (Society of Antiquities/EH Monograph, 2009).

[37] Zierden MA, Reitz EJ (2009). Animal use and the urban landscape in colonial Charleston, South Carolina, USA. Int. J. Hist. Archaeol..

[38] Lalueza-Fox C, Calderon FL, Calafell F, Morera B, Bertranpetit J (2001). MtDNA from extinct Tainos and the peopling of the Caribbean. Ann Hum Genet.

[39] Lalueza-Fox C (2003). Mitochondrial DNA from pre-Columbian Ciboneys from Cuba and the prehistoric colonization of the Caribbean. Am J Phys Anthr..

[40] Mendisco F (2015). Where are the Caribs? Ancient DNA from ceramic period human remains in the Lesser Antilles. Philos Trans R Soc L. B Biol Sci.

[41] Schroeder Hannes, Sikora Martin, Gopalakrishnan Shyam, Cassidy Lara M., Maisano Delser Pierpaolo, Sandoval Velasco Marcela, Schraiber Joshua G., Rasmussen Simon, Homburger Julian R., Ávila-Arcos María C., Allentoft Morten E., Moreno-Mayar J. Víctor, Renaud Gabriel, Gómez-Carballa Alberto, Laffoon Jason E., Hopkins Rachel J. A., Higham Thomas F. G., Carr Robert S., Schaffer William C., Day Jane S., Hoogland Menno, Salas Antonio, Bustamante Carlos D., Nielsen Rasmus, Bradley Daniel G., Hofman Corinne L., Willerslev Eske (2018). Origins and genetic legacies of the Caribbean Taino. Proceedings of the National Academy of Sciences.

[42] Greig K (2015). Complete mitochondrial genomes of New Zealand’s first dogs. PLoS One.

[43] Knapp M (2012). Complete mitochondrial DNA genome sequences from the first New Zealanders. Proc. Natl. Acad. Sci..

[44] West K (2017). The Pacific Rat Race to Easter Island: Tracking the Prehistoric Dispersal of Rattus exulans Using Ancient Mitochondrial Genomes. Front. Ecol. Evol..

[45] Robins JH (2008). Dating of divergences within the Rattus genus phylogeny using whole mitochondrial genomes. Mol. Phylogenet. Evol..

[46] Allen JA (1916). List of mammals collected in Colombia by the American Museum of Natural History expeditions, 1910-1915. Bull. Am. Museum Nat. Hist..

[47] Zúñiga, H., Pinto-Nolla, M., Hernández-Camacho, J. I. & María Torres-Martínez, O. Revisión taxonómica de las especies del genero Cavia (Rodentia: Caviidae) en Colombia. *Acta Zool. Mex* **87**, 111–123 (2002).

[48] Campos HA, Ruiz-García M (2008). Genética poblacional de cobayas de Colombia, Cavia spp.(Rodentia: Caviidae) con marcadores moleculares RAPD. Rev. Biol. Trop..

[49] Chanlatte Baik, L. Huecoid Culture and the Antillean Agroalfarero (Farmer-Potter) Period. in The Oxford Handbook of Caribbean Archaeology (eds. Keegan, W. F., Hofman, C. L. & Rodriguez Ramos, R.) 171–183 (Oxford University Press, 2013).

[50] Rodríquez Ramos R (2010). What is the Caribbean? An archaeological perspective. J. Caribb. Archaeol..

[51] Giovas Christina M. (2017). The Beasts at Large – Perennial Questions and New Paradigms for Caribbean Translocation Research. Part I: Ethnozoogeography of Mammals. Environmental Archaeology.

[52] Eeckhout P (2013). Change and permanency on the coast of ancient Peru: the religious site of Pachacamac. World Archaeol..

[53] Hofman, C. L. The Post-Saladoid in the Lesser Antilles (AD 600/800-1492). Oxford Handb. Caribb. Archaeol. Oxford Univ. Press. Oxford 205–220 (2013).

[54] Hofman CL, Bright AJ, Rodríquez Ramos R (2010). Crossing the Caribbean Sea: towards a holistic view of pre-colonial mobility and exchange. J. Caribb. Archaeol..

[55] Laffoon JE (2014). Long-distance exchange in the precolonial Circum-Caribbean: A multi-isotope study of animal tooth pendants from Puerto Rico. J. Anthropol. Archaeol..

[56] Garson, A. Prehistory, Settlement, and Food Production in the Savanna Region of La Calzada de Paez, Venezuela. Department of Anthropology PhD, (Yale University, 1980).

[57] Sýkora, A. Manejo de Recursos Faunísticos por los Pobladores del Sitio Prehispánico en Palmasola, Estado Carabobo, Venezuela. (Universidad Central de Venezuela, 2006).

[58] Rodríquez Ramos, R. Isthmo–Antillean Engagements. Oxford Handb. Caribb. Archaeol. 155 (2013).

[59] Garcia-Casco Antonio, Knippenberg Sebastiaan, Rodríguez Ramos Reniel, Harlow George E., Hofman Corinne, Pomo José Carlos, Blanco-Quintero Idael F. (2013). Pre-Columbian jadeitite artifacts from the Golden Rock Site, St. Eustatius, Lesser Antilles, with special reference to jadeitite artifacts from Elliot's, Antigua: implications for potential source regions and long-distance exchange networks in the Greater Caribbean. Journal of Archaeological Science.

[60] Curet AL, Newsom LA, DeFrance SD (2006). Cultural continuity and discontinuity in the social history of ancient Puerto Rico: The case of the Ceremonial Center of Tibes. J. F. Archaeol..

[61] LeFebvre, M. J. Animals, Food, and Social Life Among the Pre-Columbian Taíno of En Bas Saline, Hispaniola. (University of Florida, Gainesville, 2015).

[62] Reitz, E. J. & McEwan, B. G. Animals, environment, and the Spanish diet at Puerto Real. Puerto Real Archaeol. a Sixt. Spanish T. Hisp. 287–334 (1995).

[63] Zierden Martha A., Reitz Elizabeth J., Pavao-Zuckerman Barnet, Reitsema Laurie J., Manzano Bruce L. (2018). What is this bird? The quest to identify parrot remains from the Heyward-Washington House, Charleston, South Carolina. Southeastern Archaeology.

[64] Knapp M, Clarke AC, Horsburgh KA, Matisoo-Smith EA (2012). Setting the stage–building and working in an ancient DNA laboratory. Ann. Anatomy-Anatomischer Anzeiger.

