# Author Correction: Ancient DNA of Guinea Pigs (*Cavia* spp.) Indicates a Probable New Center of Domestication and Pathways of Global Distribution

**DOI:** 10.1038/s41598-020-71841-x

**Published:** 2020-09-01

**Authors:** E. Lord, C. Collins, S. deFrance, M. J. LeFebvre, F. Pigière, P. Eeckhout, C. Erauw, S. M. Fitzpatrick, P. F. Healy, M. F. Martínez-Polanco, J. L. Garcia, E. Ramos Roca, M. Delgado, A. Sánchez Urriago, G. A. Peña Léon, J. M. Toyne, A. Dahlstedt, K. M. Moore, C. Laguer Diaz, C. Zori, E. Matisoo-Smith

**Affiliations:** 1 University of Otago, Dunedin, New Zealand; 2 Stockholm University, Stockholm, Sweden; 3 University of Florida, Gainesville, FL 32611 USA; 4 Florida Museum of Natural History, Gainesville, FL 32611 USA; 5 University College Dublin, Dublin, Ireland; 6 Université Libre de Bruxelles, Brussels, Belgium; 7 University of Oregon, Eugene, OR 97403 USA; 8 Trent University, Peterborough, ON Canada; 9 Universitat Rovira I Virgili (URV), Tarragona, Spain; 10 Muséum National D’histoire Naturelle, Paris, France; 11 Institut Català de Paleoecologia Humana i Evolució Social, Tarragona, Spain; 12 Stetson University, DeLand, FL 32723 USA; 13 University of Los Andes, Bogotá, Colombia; 14 Consejo Nacional de Investigaciones Científicas y Técnicas, Buenos Aires, Argentina; 15 Facultad de Ciencias Naturales y Museo, La Plata, La Plata, Argentina; 16 School of Life Sciences and Human Phenome Institute Fudan University, Shanghai, China; 17 Instituto Colombiano de Antropología e Historia, Bogotá, Colombia; 18 National University of Colombia, Bogotá, Colombia; 19 University of Central Florida, Orlando, FL 32816-1361 USA; 20 Arizona State University, Tempe, AZ 85281 USA; 21 University of Pennsylvania, Philadelphia, PA 19104 USA; 22 Baylor University, Waco, TX 76798 USA

Correction to: *Scientific Reports* 10.1038/s41598-020-65784-6, published online 01 June 2020

This Article contains errors. The Acknowledgements section in this Article is incomplete.

“We would like to thank Matthew Smith for providing the Green Castle, Jamaica specimen; Kitty Emery and the Environmental Archaeology Program Laboratory at the Florida Museum of Natural History for facilitating access to guinea pig specimens from Finca Valencia, Puerto Rico (Catalog #0547); William Keegan and Corinne Hofman for analysis of specimens from Giraudy, Saint Lucia; Daniel S. Sandweiss for permission to analyze the Lo Demás specimens housed at the Florida Museum of Natural History and site information; Ana Maria Boada for providing samples from El Venado, Colombia; and Martha Zierden (on behalf of Charleston Museum) and Elizabeth Reitz for providing the Charleston sample, site information and comments on the manuscript. Kuelap samples were selected and exported legally according to the Ministry of Culture of Peru Resolución Viceministerial no. 095-2015-VMPCIC-MC (August 2015). We thank Susan Duser for assistance in creating Figs. 1A and 2. We acknowledge Peter Stahl for his review of the manuscript prior to publication. Funding for this research was provided to EM-S by the Department of Anatomy, University of Otago. Open access funding provided by Stockholm University.”

should read:

“We would like to thank Matthew Smith for providing the Green Castle, Jamaica specimen; Kitty Emery and the Environmental Archaeology Program Laboratory at the Florida Museum of Natural History for facilitating access to guinea pig specimens from Finca Valencia, Puerto Rico (Catalog #0547); William Keegan and Corinne Hofman for analysis of specimens from Giraudy, Saint Lucia; Daniel S. Sandweiss for permission to analyze the Lo Demás specimens housed at the Florida Museum of Natural History and site information; Ana Maria Boada for providing samples from El Venado, Colombia; and Martha Zierden (on behalf of Charleston Museum) and Elizabeth Reitz for providing the Charleston sample, site information and comments on the manuscript. Kuelap samples were selected and exported legally according to the Ministry of Culture of Peru Resolución Viceministerial no. 095-2015-VMPCIC-MC (August 2015). We thank Susan Duser for assistance in creating Figs. 1A and 2. We acknowledge Peter Stahl for his review of the manuscript prior to publication. Funding for this research was provided to EM-S by the Department of Anatomy, University of Otago. Open access funding provided by Stockholm University. We would like to thank Marceline Denis and Cécile Ansieau (on behalf of the Walloon Heritage Agency) for providing the specimen from Mons, Belgium.”

Additionally, there are typographical errors. In the legend of Figure 2,

“The routes depicted are purely hypothetical; people may have translocating guinea pigs using routes other than those depicted.”

should read:

“The routes depicted are purely hypothetical; people may have translocated guinea pigs using routes other than those depicted.”

In the Author Contributions section,

“E.L., E.M.-S., S.d.F., M.J.L. conceived the study. E.L. carried out the laboratory work and analyses with assistance from C.C., S.dF., M.J.L., F.P., P.E., C.E., S.M.F., P.F.H., M.F.M.-P., J.L.G., E.R.R., M.D., A.S.U., G.A.P.L., J.M.T., A.D., K.M.M., C.LD., and C.Z. provided samples.”

should read:

“E.L., E.M.-S., S.d.F., M.J.L. conceived the study. E.L. carried out the laboratory work and analyses with assistance from C.C.. S.dF., M.J.L., F.P., P.E., C.E., S.M.F., P.F.H., M.F.M.-P., J.L.G., E.R.R., M.D., A.S.U., G.A.P.L., J.M.T., A.D., K.M.M., C.LD., and C.Z. provided samples.”

