# Supplementary material for: Ancient DNA of Guinea Pigs (Cavia spp.) Indicates a Probable New Center of Domestication and Pathways of Global Distribution
Source: Sci Rep. 2020 Jun 1;10:8901. doi: 10.1038/s41598-020-65784-6 (PMC7264122; doi:10.1038/s41598-020-65784-6)
Supplement: Supplementary file 1 — Supplementary Information. [file 41598_2020_65784_MOESM1_ESM.docx]

Supplementary Information for

**Ancient DNA of Guinea Pigs (*Cavia* spp.) Indicates a Probable New Center of Domestication and Pathways of Global Distribution**

Lord, E.^a,b*^, Collins, C.^a^, deFrance, S.^c^, LeFebvre, M. J.^d^, Pigière, F.^e^, Eeckhout, P. ^f^

Erauw, C.^f^, Fitzpatrick, S. M.^g^, Healy, P.F.^h^, Martínez-Polanco, M. F.^i-k^, Garcia, J. L.^l^, Ramos Roca, E.^m^, Delgado, M.^n-p^, Sánchez Urriago, A.^q^, Peña Léon, G. A.^r^, Toyne, J. M.^s^, Dahlstedt, A.^t^, Moore, K. M.^u^, Laguer Diaz, C.^c^, Zori, C.^v^, & Matisoo-Smith, E.^a*^

Email: edana.lord@zoologi.su.se or lisa.matisoo-smith@otago.ac.nz

**This PDF file includes:**

Supplementary text

Figs. S1 to S4

Tables S1 to S6

References for SI

Supplementary Information Text

**1. Samples and Methods**

1.1 Archaeological Site Descriptions and Specimen Context

We provide site descriptions for all sites with ancient samples used in the phylogenetic analyses.

**Caribbean Archaeological Sites**

***Tibes, Puerto Rico***

The site of Tibes is a large ceremonial center (~16 ha) located on the Portugués River near the south coast of Puerto Rico. Sometime between AD 300-400, a small village was founded by tropical horticulturalists who also hunted, fished, and collected a variety of shellfish from the coast approximately 8 km away. These initial colonists are associated with the Saladoid culture, horticulturalists who migrated from South America into the Caribbean beginning at ~500 BC. Tibes underwent major architectural and spatial reorganization between AD 900-1200 when communal plazas, ballcourts, and causeways were built and a ranked society is believed to have emerged. This political and social transformation from a simple village to a civic-ceremonial center occurred during the subsequent Ostionoid culture period (AD 600-1200) (see [1,2]). Tibes is the earliest known civic center in the Greater Antilles. Guinea pigs are interpreted as food remains from a broad diet of marine, riverine, and terrestrial fauna (see [3,4]). Both of the guinea pig mandibles selected for aDNA analysis are from deposits dating to the Ostionoid time period (AD 600-1200). The specimens selected for aDNA analysis are from Tibes 1996, Unit 7, Level 2, Bag #264 (Sample Tibes A) and is relatively dated to AD 600-900. The second specimen (Tibes B) is from Tibes 2003, Unit N184 E55, Level 5, Bag #1376, #179 and relatively dated to AD 900-1200.

***Finca Valencia (NCS-1), Puerto Rico***

The Finca Valencia site, also known as NCS-1, is also located in Puerto Rico within the northern coastal plain. Archaeological evidence indicates the site was occupied during the Saladoid (ca. 500 BC-AD 500) and Ostionoid (AD 500 - 1500) pre-Columbian periods, as well as the late 18^th^-19^th^ centuries. The Saladoid component of the site is characterized as a small temporary settlement without connection to later pre-Columbian occupations. Radiocarbon dates indicate that the most concentrated period of pre-Columbian occupation was during the Ostionoid period, beginning ca. AD 1200 and continuing into the 15^th^ century [5]. During this time, Finca Valencia was a substantial village site composed of several structures, burials, and at least one ball court.

Originally analyzed by Elizabeth Wing, the guinea pig assemblage from Finca Valencia is the largest recovered from the Caribbean to date [6]. The remains are interpreted as likely subsistence, as there are no archaeological indications of ritual significance attached to the specimens [7]. The guinea pig mandibular specimens selected for aDNA analysis are associated with a conventional radiocarbon age of 530 +/- 140 BP or cal AD 1192-1792, (IntCal 13, [8]) ([5]: Table 29). The sample contexts are: NCS -1A Unit N2260 E780, Strata II, Level 5; NCS -1B Unit N2260 E780, Strata II, Level 7; NCS -1C Unit N2270 E780, Strata II, Level 5; NCS -1D Unit N2260E780, Strata II, Level 5. The specimens are curated in Environmental Archaeology, Catalog number 0547, at the Florida Museum of Natural History, Gainesville

***Grand Bay, Carriacou***

Grand Bay was a large Amerindian village situated on the southeastern (windward) coast of Carriacou in the southern Grenadines. Archaeological investigation of the site over seven field seasons between 2003-2014 demonstrated that it covered an extensive area of at least 6000 m^2^ and was occupied intensively for nearly a millennium between ca. AD 400-1300 [9] spanning the terminal Saladoid to late Suazan (Ostionoid) periods. Remnants of past human activities include residential structures (postholes), dense midden deposits with an array of vertebrate and invertebrate remains [9,10], numerous human burials, and refuse pit features. Petrographic and chemical analysis of ceramics [11] and evidence for translocation of at least four mammals (armadillo, opossum, agouti, and guinea pig, as well as importation of deer artifacts and elements [12,13], testify to widespread movement and exchange of material goods between Carriacou and other islands and the South American mainland. The guinea pig sample used in this study comes from one of four elements (posterior halves of a left and right zygomatic arch and complete, articulating left and right maxillary halves) from a single individual recovered from Trench 446, Square 9, Layer 002, Planum 4 (Sample No. 05CGB000609) in deposits that contained Late Ceramic Age (Suazan) pottery. This association was confirmed by dating one of the maxillary specimens by AMS to cal AD 985–1030 (1020 ± 20 yr BP; UCIAMS-94045).

***Coconut Hall (PE-15), Antigua***

The Coconut Hall site, also known as PE-15, is located on a peninsula-like point of low-lying bedrock on the east coast of Antigua. A small littoral site, it lies between Mercers Creek Bay to the east, and Guiana Bay to the north, at elevations that range from 0 to 21masl. Surface trace remains of several shell midden clusters, consisting of food refuse remains (e.g., bone, shell, crab), as well as prehistoric household artifacts (e.g., stone, shell, pottery) were noted in 1993 [14]. Excavation revealed a coastal shell midden representing, at least in part, a prehistoric settlement and subsistence-processing site. Archaeological evidence indicates that the Coconut Hall site was occupied during the Post-Saladoid (Mamoran-Troumassoid series) pre-Columbian period of the Lesser Antilles, ca. AD 900-1200 [15]. The presence of ceramic griddle fragments suggests some subsistence reliance on manioc (Olsen 1974:37-43). However, based on a detailed faunal analysis (NISP = 3117; MNI = 246), it is evident that the Coconut Hall inhabitants also relied quite heavily on marine resources ([16]: Table 2). Terrestrial habitat species (birds, reptiles, and mammals) compose less than one-quarter of the vertebrate fauna. Among these, guinea pig was a noteworthy mammalian specimen, presumably exploited as a supplementary source of food [6]. The guinea pig mandibular specimen from Coconut Hall selected for aDNA analysis is associated with two conventional radiocarbon dates of 1370+/-60 BP and 1350+/-60 BP ([16]: Table 1) and a calibrated date range of Cal AD 930-1180 and Cal AD 945-1190. The specimen, recovered from Excavation 1, Stratum F3-6 (0-40 cm), is curated today in Environmental Archaeology, Lab ID MS10630, at the Florida Museum of Natural History (FMNH).

**Colombian Archaeological Sites**

***El Venado, Colombia***

El Venado is a settlement located in the Samacá Valley, in the northeastern highlands of the Colombian Andes. An ephemeral occupation by a small group of people with a radiocarbon date of 2645 ± 125 BP (GX-22394; δ^13^C= -23.4 ‰ ]) left the remains of a small base camp with a fire pit and lithic debitage. Later on, around AD 800, (Herrera Period) a small agricultural community developed in the site forming two distinctive residential wards 100-150 m apart with evidence of some degree of social hierarchy based on remains of feasting and ceremonialism, concentrated at la Esmeralda ward. By the next period, the Early Muisca (AD 1000 -1250), the two previous wards expanded and two new wards were founded. Social hierarchy at this time seems to have been based on economic wealth, through the specialized production of textiles at la Esmeralda ward. There is also evidence of feasting and ceremonial functions carried out at La Esmeralda ward. During the Late Muisca period (AD 1250-1600), the El Venado settlement grew and a new ward was added. Social differentiation was mostly based on wealth. Although feasting and ceremonies continued to be carried out at La Esmeralda ward, evidence of feasting began to appear in another ward, maybe symptomatic of intra-site competition among residential groups. Faunal remains, identified by Elizabeth Ramos, indicated the consumption of deer, domesticated guinea pig (*Cavia porcellus*) and wild guinea pig (*Cavia aperea*) and undetermined guinea pig (*Cavia* sp.) as the main source of protein; although other species such as spotted cavi *(Agouti paca* and *Agouti taczanowskii)*, armadillo (*Dasypus novemcinctus* or *D. kappler)*, and coatimondi (*Nasua* sp.) were also consumed occasionally throughout the archaeological sequence of El Venado [17].

The sample of guinea pig remains from El Venado come from midden deposits and human burials. The samples contexts are: Sample 257 Test Pit 5, 333E 280N, Level 4, partial mandible, Late Herrera period; Sample 258 Test Pit 5, 633E 280N, Level 5, partial cranium, Late Herrera period; Sample 260 Test Pit 6, 649E 249.5N, Tomb-12, partial mandible, Late Muisca period; Sample 261 Test Pit 6, 649E 249.5N, Tomb-12, partial mandible, Late Muisca period; Sample 262 Test Pit 56, 649.1E 287.7N, Tomb-20, partial mandible Early Muisca; Sample 265 Test Pit 5, 635E 280N, Level 5, partial mandible, Late Herrera period.

***Madrid (2-41), Colombia***

The Madrid 2-41 site is located in Cundinamarca, Colombia within the Bogotá high plain. The Bogotá high plain is a well-known archaeological region located in the eastern Cordillera of Colombia, northern South America, where important evidences of human occupation have been recovered for a period that encompasses more than 12,000 years (Delgado 2012). Archaeological evidence indicates the site was used mainly during the Herrera period (*ca.* 300 BC – AD 900), specifically during the early (*ca.* 2000 years B.P) and late (AD 900) phases. In addition, the site also was used during the Muisca (*ca.* AD 1000-1600) and colonial periods (ca. AD 1700). The Madrid site corresponds to a large ceremonial site where individual and collective burials with several human remains along with abundant archaeological materials that includes pottery, lithic artifacts, faunal remains among others were archaeologically recovered [18,19]. Two main contexts were found, one characterized by several burials with 11 human skeletons and faunal remains (including deer and guinea pig) related to the early Herrera phase associated to a conventional radiocarbon date 2100 ± 50 (Beta-204120) and a ceremonial context which presents multiple circular features with several offerings, three human skeletons and animal remains related to the late Herrera phase 1220 ± 40 years B.P (Beta-259738). Since the guinea pig remains included in the analysis were found in a feature of the early context near the human burials they are interpreted as subsistence fare belonging to the early Herrera phase. The guinea pig mandibular specimen selected for aDNA analysis is from unit 1, cut 5 (90-110 cm deep) of the early burial context.

***Aguazuque I, Colombia***

The Aguazuque archaeological site is located in the ‘hacienda’ named after it, in Soacha, Cundinamarca in the south of Bogota, Colombia. This site was excavated by Gonzalo Correal in the 1980’s. Correal [20] identified six different human occupations at Aguazuque, with chronologies ranging from 3000 to 1000 BC. Five of the occupations were preceramic, and the sixth contained ceramics from different chronologies, indicating a mixed deposit. According to Correal [20] the occupations were enduring and it is possible that they were product of dense human group occupations. Preceramic occupations are characterized by the Abriense lithic industry, mainly used to process and prepare hunted animals. However, other kind of lithic artifacts were also found, such as milling stones for vegetable processing [20].

In occupation 3, circular structures covered with red ocher were discovered. There was evidence of fire over these structures. Associated faunal remains and other materials are white-tailed deer (*Odocoileus virginianus*) and guinea pigs (*Cavia* sp.) as well as sandstone [20]. Evidence of vegetable handling including squash (*Curcubita pepo*) and oca (*Oxalis tuberosa*) was identified in occupation 3 [20]*.*

Aguazuque is an open air site in a strategic geographical position close to swamps and wetlands that are relics of a large lake that covered the Sabana de Bogota during the Pleistocene era. Given the location of the site, it was possible that the inhabitants hunted animals such as white-tailed deer and guinea pigs. At the same time, Aguazuque was relatively closed to the Magdalena River; therefore, the occupants could use other faunal resources [20].

Martínez-Polanco [21]studied the guinea pigs remains from Aguazuque. In this zooarchaeological and taphonomical analysis it was determined that guinea pigs were a very important part of human diet in Aguazuque. This interpretation of food use is supported by anthropogenic modifications related to the butchering, processing, and consumption of guinea pigs. The guinea pig samples are associated with the third occupation of the site (Capa 4.2) with a date of 1885-1815 BC.

***Checua, Colombia***

Checua is an open air archaeological site near a rock shelter located in the Nemocón municipality to the north of Bogotá. Checua was excavated by Ana María Groot in 1991 [22]. This site is a hunter-gatherer settlement characterized by the presence of Abriense lithic artifacts and faunal remains of white-tailed deer and guinea pig *(Cavia* spp.) with a chronology ranging from 6,000 to 1,000 BC, separated into four occupations [22]. The first occupation is interpreted as an occasional camp site. The second represents more frequent and prolonged occupations. In the third occupation there is a round structure 3.5 m that was probably a domestic structure. The fourth occupation represents a more intensive human occupation [22]. The guinea pig samples selected come from the second occupation of the site (5940-5760 BC) (Beta -53924, CH-1). The specimens include are mandibles (LAB-ARQ-UN-524-C2-60-65 cm. LAB-ARQ-UN-840-C2-70-75 cm. LAB-ARQ-UN-2045-C2-85-90 cm.), incisive bones (LAB-ARQ-UN-530-C2-60-65 cm.) and isolated molars (LAB-ARQ-UN-2055. C2-85-90 cm).

**Peruvian Archaeological Sites**

***Kuelap, Peru***

The monumental complex of Kuelap is located at 3000 masl in the Utucbamba River valley in the Department of Amazonas, Peru. It was constructed atop a massive masonry platform along a ridgeline and measures almost 700 m long by 150 m wide. With over 400 house structures and a key ritual temple, it is a densely occupied urban complex [23]. The earliest occupation appears to have established the site as a religious center in approximately AD 500, but the majority of the residential construction occurred between AD 900-1470 under the Chachapoya culture and later modified only slightly under Inca rule from AD 1470-1535 [24]. The houses do not dramatically show architectural variation to suggest internal hierarchical social complexity, leaving archaeologists to argue that Chachapoya society may have been organized as chiefdoms with various regional autonomous groups who created a confederacy against the Inca invaders [25,26]. They were primarily agro-pastoralists with a wide range of domesticated crops that ranged along the terraced slopes between 1750 and 4300 masl including potatoes, maize, quinoa, and beans. Faunal remains indicate that while llamas were commonly consumed, other wild species such as deer and larger rodents were also regularly eaten. A long, narrow masonry tunnel at the base of the bench within most houses has been interpreted as a domesticated guinea pig corral or run, suggesting that they were kept in individual houses in large quantities, primarily as food. However, faunal recovery has produced a limited number of guinea pig skeletal remains. Stable isotope analysis of guinea pig diet show they were opportunistically provisioned, eating grasses but supplemented with a range of household scraps (Toyne, personal communication 2018). The specimens selected for aDNA are primarily from House Structures 1 and 2 in the southern end of the site near the Templo Mayor. Specific radiocarbon dates are not available for each specimen or context, but most were from superficial excavations suggesting they probably date to the later occupation (AD 1100-1535) of the site.

***Pachacamac, Peru***

Pachacamac is a 500-plus hectares large monumental settlement located at the mouth of the Lurín River, on the Central Coast of Peru. It has been occupied from the 5^th^ to the 16^th^ Centuries AD as shown through successive Lima, Wari, Ychsma, Inca and Early Colonial evidence excavated and C14 dated materials from the site [27]. The Sacred Precinct contains the major temples that made the site famous, while the Second Precinct shows a proto-urban design with elite residences, minor temples, streets, plaza, cemeteries and others structures [28]. The Third Precinct, almost unexplored, is probably where most of the common people were living.

In total, nine guinea pig mandibular samples originating from different contexts and periods were excavated by *The Ychsma Project (ULB)* within the Second Precinct. Sample 1 comes from a cemetery dating to the end of the Middle Horizon to Late Intermediate Period (LIP, ca AD 1000-1470) [29]. Samples 2 and 3 are from the same period but from a domestic layer maybe related with the ancestor cult in building B15 [30]. Samples 4, 5 and 6 date to the end of the LIP and were found in domestic contexts. Finally, samples 7, 8 and 9 date from Late Horizon to Early Colonial Period (ca. AD1470-1572) and are from a sector with a ritual function within building B15 [31]. The contexts of the samples are: Pachacamac 1 - P13-U100-a-4 (2012); Pachacamac 2 - B15-U126-a-4 (2014); Pachacamac 3 - B15-U126-a-4 (2014); Pachacamac 4 - P12-U123-a-4 (2014); Pachacamac 5 - P12-U123-a-5 (2014); Pachacamac 6 - P12-U123-a-6 (2014); Pachacamac 7 - B15-U124-f-1 (2014); Pachacamac 8 - B15-U124-i-1 (2014).

***Moqi, Peru***

Moqi is a single-occupation Late Horizon (AD 1400-1532) site located in the Locumba Valley of southern Peru. This site appears to have been constructed at the order of the Inca given the absence of pre-Inca habitation and rapid abandonment after the fall of the empire. Measuring just over 25 ha in size, Moqi can be divided into two sectors: the Inca civic-ceremonial center of Moqi Alto and a zone of local habitation designated as Moqi Bajo. Moqi Bajo includes both a domestic sector and an artificially flattened hilltop with a plaza surrounded by rectangular buildings that approximate Inca style and construction techniques. The site was intentionally abandoned sometime between AD 1510 and 1600, corresponding to the weakening of the Inca Empire [32].

The site includes six cemeteries of subterranean, primarily stone-lined, cist tombs. Each tomb held between one and eight individuals, with burial offerings including combs, wooden spoons and boxes, weaving equipment, local and Inca style ceramics, and guinea pig remains. Of the five guinea pig specimens selected for aDNA analysis, four are from tombs in Cemetery 2 (TP4-F03-H03, TP6-F02-H01, TP6-F02-H03, TP6-F02-H07) and one was interred on top of a basket in Moqi Bajo (L36-F02). Radiogenic strontium isotope (^87^Sr/^86^Sr) analysis indicates all analyzed guinea pigs were raised locally [32].

***Lo Demás, Peru***

Lo Demás is an archaeological site located in the Chincha Valley on the Peruvian coast about 200 km south of Lima. Artifactual and radiocarbon data indicate that Lo Demás dates to the brief period when Chincha was part of the Inca Empire (the Late Horizon), from approximately AD 1480 -1540 [33]. The site was occupied by fisher families in a commoner sector and fishing lords in a monumental sector. Ethnohistoric documents suggest that the site was part of a much longer, coast-parallel fishing settlement [34]. The monumental sector provided evidence for craft production (cotton and gourds) under the aegis of the fishing lords, while the remains from the commoner sector supported the suggestion from the early documents that the inhabitants were specialized fishermen. Excavations produced abundant evidence of guinea pig use throughout the site: “1) feces; 2) disarticulated bones, often charred or gnawed; and 3) naturally mummified remains of whole guinea pigs” ([35]:51-52). The first two categories were found throughout the site, while the whole guinea pigs came from Sector I (commoner residences) and Sector II (a burial zone). One of the Sector I guinea pigs had had its stomach slit open, just as curanderos do today when they use guinea pigs as a medical diagnostic device [33,35]. This find indicates that animal divination using guinea pigs is a pre-Columbian practice.

***Torata Alta, Peru***

Torata Alta is a late pre-Columbian and Spanish colonial settlement located in the Torata Valley of far southern Peru in the Department of Moquegua. The site is interpreted as having been settled initially by the Inca as an agricultural village during late pre-Columbian times [36]. During the second-half of the 16^th^ century the settlement was transformed into a *reducción* - a locale where native Andean peoples were forcibly relocated to facilitate Spanish colonial control and religious conversion. Under Spanish rule, the settlement was arranged on a gridded street layout and included a possible plaza and a church. Indigenous ceramics and other material good suggest that that the resettled populations originated from the Lake Titicaca region and were affiliated with Lupaqa peoples [37]. The economic activities of the site included agricultural production and textile manufacture, the latter of which intensified under Spanish control and probably functioned to provide cloth as a form of tribute to Spanish overlords [37].

The most extensive excavations at the site were completed under the auspices of the Moquegua Bodegas Project [37]. Complete and partial excavations of several structures recovered primarily domestic remains associated with indigenous households living under Spanish control. Trench excavations outside of structures revealed refuse discarded outside of structures. The site was occupied at the time of the February 1600 eruption of the Huaynaputina volcano as indicated by the presence of ash-fall with the excavated structures. The site was reoccupied following the volcanic eruption; however, the site was abandoned during the 17^th^ century when villagers relocated to other parts of the Torata Valley. Although the site may have been important during Inca rule of the region and strategic for Spanish rule [36], the occupation was relatively short-lived.

Guinea pig remains were only found in trench excavations [38]; however, excavations revealed interior domestic spaces that are interpreted as guinea pig pens [37]. The guinea pig specimen included in the analysis is a sub-adult individual from a pre-1600 (below the volcanic ash) trench excavation (Trench 6) that contained domestic refuse.

**Bolivian Archaeological Sites**

***Chiripa, Bolivia***

The site of Chiripa, dominated by a Late Formative period mound and sunken temple, was the center for earlier village and ceremonial centers on the Taraco peninsula of Lake Titicaca, Bolivia. The Llusco sector of the site is the third of multiple rectangular sunken plazas thought to be the location of early social integration and feasting [39]. During the occupation of the Llusco area, a mixed economy of horticulture, camelid pastoralism, fishing and foraging for wild plants and animals in the nearby wetlands provided the subsistence base [40–42].

The samples selected for analysis were from Locus 67 and 68 within the Llusco structure. They are from hand-collected samples noticed by excavators as concentrations of rodent bones. In both samples, remains of at least two guinea pigs were encountered in a discrete area, suggesting they may have been in a deliberate deposit. Apart from the remains of these small mammals, the sediments were identified as midden used to fill the Llusco sunken court, a semi-subterranean structure approximately 11m by 13m. The fill contained concentrations of mammal and fish bone, burned earth with plant impressions, and ash, suggesting a secondary context for most of the remains. Radiocarbon dates for the Llusco structure indicate that this material dates from the Late Chiripa Phase (Middle Formative), approximately 800-750 BC [43].

***Kala Uyuni, Bolivia***

The Kala Uyuni (KU) site is a complex of Formative structures and surfaces facing Lake Titicaca, on the southern side of the Taraco Peninsula. The lower part of the site is consists of houses with cobble masonry footings, courtyards and walls (the KU sector). The guinea pig samples were chosen from midden areas to the southwest of the KU sector, in an area known as Kala Uyuni Ayrampu Qontu (KU AQ). These KU AQ deposits are domestic midden, possibly representing an area of production and preparation for larger communal activities in the central part of the site [44,45]. Guinea pig remains came from Locus units 5061 and 5072, layers in stratified deposits containing mammal and fish bones, ceramics, and lithics. The matrix consisted of cobbles and patchy clay, leading excavators to speculate that the material was in close association with eroded mud brick walls or surfaces. In both loci, multiple individual guinea pigs were collected in ¼” (6.35 mm) sieves, along with other food remains. Both samples were associated with Late Chiripa phase ceramics, dated to between 900 and 700 BC [45]. Detailed paleoethnobotanical and zooarchaeological analysis shows that the agricultural system of Late Chiripa (Middle Formative) times had not yet resulted in the intensive agriculture and land disturbance documented in the Late Formative [41,46]. Wetland resources would have been important for food and craft material.

**European Archaeological Site**

***Mons, Rue Jean Lescarts, Belgium***

The ‘Rue Jean Lescarts’ site is located in the town of Mons, Belgium (Province of Hainaut). The excavations revealed a living quarter at the margin of the town centre, dating from medieval times until today [47]. In the 16th and 17th centuries, an increased urbanisation occurred and habitation by both middle and lower class can be observed: market halls, abbey shelters, notable’s residences and modest houses coexist in the same sector [48]. During the Renaissance, under the Spanish domination, the town of Mons experienced a relative increase in prosperity due to local production of high quality items and commercial exchange [49].

In total, eight guinea pig bones have been discovered at this site that all belonged to a single individual [48]. Six of the bones were found in the fill of a cellar while the remaining two came from an adjacent cesspit that was located in the backyard of a middle class residence. The cesspit was constructed after the cellar and partially dug into it. The boundary between the two contexts was not always clear during the excavation, which explains why some material from the cellar may have ended up in the cesspit material. It is most probable that all the guinea pig bones originated from the cellar. None of the bones show traces of processing. Taking into account the excellent state of preservation of the bones and the completeness of the various skeletal elements, these remains can be safely considered to represent a carcass rather than food refuse.

The high quality of numerous artefacts discovered in these two contexts seems to indicate a quite privileged citizen. The context contained armorial sandstones, majolica tableware, ‘façon de Venise’ glasses and painted stained-glass windows. Based on this archaeological material, the fill in which the guinea pig remains were found dates to the end of the 16th - beginning of the 17th century. In order to confirm the dating of the remains of the animal, the left tibia was submitted for radiocarbon dating that yielded an age of 370+25BP (KIA-43023) or cal. AD 1440-1530 (57.8%) and AD 1550-1640 (37.6%) [48]. The right tibia selected for aDNA analysis is from the same archaeological context MN07JLE US 4002.

**North American Archaeological Site**

***Heyward-Washington House, Charleston, South Carolina***

The Heyward-Washington site (38Ch108) is a residential property located in Charleston, South Carolina. The ca. 1772 house and outbuildings are operated as a historic house museum by The Charleston Museum, who has owned the property since 1929. The lot at 87 Church Street is located within the walled city, the section of Charleston settled by the turn of the 18^th^ century. Gunsmith John Milner built a small wood house and sheds, forges, and wells for the business in the 1730s, although these were demolished by Charleston’s great fire of 1740. His son, John Milner Jr. built a brick house and service buildings in 1749. Planter Thomas Heyward purchased the property in 1770 and replaced the brick single house with a larger Georgian-style home, which President George Washington rented the house during his 1791 southern tour. The property served as a planter’s home, boarding house, and bakery through the 19^th^ century. The property currently features the c. 1772 main house and privy, c. 1749 kitchen and stable buildings, and formal garden re-established in 1930. Elaine Herold of The Charleston Museum conducted extensive excavations at the site in 1973-1976, including the kitchen, work yard, main house cellar, and privy. Martha Zierden excavated the stable building in 1991 and 2002. Elizabeth Reitz analyzed faunal remains from the stable excavations in 2003. Level 6, dating to the first quarter of the 19^th^ century, produced the remains of the guinea pig, as well as a parrot (identified as a blue-fronted Amazon*, Amazona aestiva,* see Zierden et al. [50]). Remains from the guinea pig included the skull, two innominates, humerus, femur, and tibia. The femur was selected for aDNA analysis. The faunal collections are curated at The Charleston Museum, under accession #1978.38 and catalog number ARL 19915.

**Modern Sample**

***San Sebastián Puerto Rico***

The modern Puerto Rican sample was obtained for comparative purposes from a farmer’s market located at the Plaza Agropecuaria de San Sebastián, in the town of “San Sebastián de las Vegas del Pepino” (San Sebastián for short). The Plaza Agropecuaria is the most recent iteration of the farmer’s market in the town. Individuals from the surrounding towns (from the coast to the mountainous interior) meet weekly, starting with the auction of cattle and fresh meat vendors at dawn, and continuing throughout the day with a variety of staple products and goods.

Eating guinea pig on the island is uncommon, and although it is not illegal to eat it, it is illegal for most vendors to sell the meat. The guinea pig specimen was advertised as a pet alongside other guinea pigs. The specimen was a male with black fur, and locally bred. The vendor is a breeder of a variety of farm animals on the coastal town of Hatillo.

1.2 Supplementary Methodology

**Sample preparation**

All archaeological samples were processed in a purpose built ancient DNA facility at the University of Otago [51]. To remove surface contamination, samples were immersed for 30 seconds in 5% bleach and rinsed twice in ultrapure water. Samples were dried under UV light overnight.

### Ancient DNA extraction and Library preparation

Approximately 50-250mg of each archaeological sample was ground to a fine powder using a sterile mortar and pestle. DNA extraction was undertaken following Rohland and Hofreiter [52]. Double stranded libraries were prepared from the DNA extracts for sequencing on an Illumina platform following Matisoo-Smith et al. [53].

### Hybridsiation capture and sequencing

Hybridsation capture of the complete mitochondrial genome was undertaken following Marcic et al. [54] with modifications as per Grieg et al. [55]. Bait DNA was prepared from modern guinea pig tissue as described in Lord et al. [56]. Captured libraries were eluted in 20μL TE, pooled in equimolar ratio and sent for sequencing on a 2 x 75 bp paired end run on an Illumina MiSeq at the Otago Genomics and Bioinformatics Facility at the University of Otago, Dunedin, New Zealand.

### Bioinformatic analyses

Raw reads were processed through an in-house pipeline described below. Adapters and low quality reads were removed from the FASTQ files using AdapterRemoval v2 [57]. In order to assess possible contamination the sequences were then aligned to a composite reference sequence guinea pig (*Cavia porcellus*, Genbank ID NC_000884.1), human (*Homo sapiens*, NC_012920.1), pig (*Sus scrofa*, NC_00845.1), chicken (*Gallus gallus*, NC_001323.1), domestic dog (*Canis lupus familiaris*, NC_002008.4), cow (*Bos taurus*, NC_006853.1) and Pacific rat (*Rattus exulans*, NC_012389.1) using BWA (v0.7.15) with recommended parameters for ancient DNA (−n 0.03 –o 2 –l 1024) [58]. Duplicate reads were removed using Picard’s MarkDuplicates [59]. Map Damage (v2.0) was used to assess if the patterns of DNA damage associated with ancient DNA were present and appropriate for the age and context of the samples [60]. Variant call files (VCFs) were produced using GATK’s HaplotypeCaller (v3.5) [61]. Consensus sequences were produced from the VCFs, with a read depth of two required to call the variants. For samples with an average read depth of less than 10, consensus sequences were produced with a read depth of one required to call the variants. Coverage per base was calculated using Samtools [62] and plotted for each sample from the BAM files, using ggplot in R v3.5.3 [63,64].

#### De novo assembly

All samples from Colombia failed to map efficiently to the domestic guinea pig reference mitogenome (Genbank: NC_000884.1) (see SI Fig S1), thus a de novo assembly was carried out using the highest coverage Colombian sample (MS10677). Adapters were removed and low quality reads trimmed using AdapterRemoval v2 [57]. Reads were then imported into Geneious (v11.0.4) and a de novo assembly was performed using Geneious with the default parameters, and the circular contig option [65]. The contigs were then mapped to the *Cavia porcellus* mitochondrial reference genome (Genbank: NC_000884.1), with the option to dissolve and reassemble contigs selected. A consensus sequenced was produced using a mapping quality of 30, and read depth greater than two. The consensus sequence was manually edited to remove any ambiguous bases (R or K, replaced with N) and then used as the reference to align the remainder of the Colombian mitogenomes as described above. Consensus sequences were produced from the VCFs with a read depth greater than two.

To determine the authenticity of the de novo assembly, a *C. porcellus* sample (MS10625) was aligned to the Colombian reference (MS10677), as described above. This showed showed similar inefficient mapping as seen previously when the Colombian samples were mapped to the *C. porcellus* reference.

**Phylogenetic Analyses**

The consensus FASTA sequences were imported into Geneious and aligned with the addition of the modern reference sequence (Genbank: NC_000884.1) using MUSCLE [65]. PopArt was used to produce a Median Joining haplotype network [66] from the alignment of all the complete mitogenome sequences. A custom python script was used to add a traits block to the alignment in order to colour the haplotypes by geographic location. The Cytochrome B region was extracted from the complete mitogenome alignment and aligned with 115 sequences from modern *Cavia* species, and Caviidae outgroups provided on Genbank [67–72] (SI Table S5). Maximum likelihood and Bayesian analyses were undertaken to analyze the relationship between the *Caviidae* species, using IQTree and BEAST v2.4.7 respectively [73,74]. The evolutionary model was determined to be HKY+I+G using jModelTest (v2.1.10) [75]. For IQtree, 1000 bootstrap replicates were undertaken, and the evolutionary model was also determined to be HKY+I+G using the built in model test. For the Bayesian analyses, the HKY+I+G model was selected, with a strict clock and the Birth Death model tree prior selected. The analysis was run for 10 million generations, sampling at every 1000 generations. Tree Annotator [76] was used to remove 10% burnin of the Bayesian tree and both Bayesian and ML trees were visualised in Figtree v1.4.3 [77]. The phylogenies produced by both the ML and Bayesian approaches were identical, thus a single tree with both bootstrap and posterior probabilities is displayed.

Fig. S1. Coverage plots of read depth across the complete mitogenome for a representative Peruvian sample (MS10681) and the Colombian samples (MS10639, MS10672, MS10675 and MS10677) mapped to the *C. porcellus* reference (Genbank ID: NC_000884.1).

MS10639 (100%)

MS10672 (100%)

MS10675 (100%)

Fig. S2. Coverage plots of read depth across the complete mitogenome for the Colombian samples (listed in Figure S1) mapped to MS10677 (post de novo assembly).

Fig. S3. Coverage plot showing read depth across the mitogenome genome for sample MS10625 (*C. porcellus* from Tibes, Puerto Rico) mapped to sample MS10677 (Colombia) (post de novo assembly).

Fig S4. Maximum Likelihood Tree for Cytochrome B from Cavia Species. Bootstrap and posterior probability support above 80/0.8 respectively is shown.

Table S1. Context and description of all samples analyzed in the study (n=66 archaeological samples and two modern Guinea pigs).

|  | **Sample** | **Region** | | **Site** | **Age** | **Element (side)** | **Reference** |
| --- | --- | --- | --- | --- | --- | --- | --- |
| 1 | MS10708 | Caribbean | | Green Castle, Jamaica | Post AD500 | Femur proximal ¼ (R) | [78] |
| 2 | MS10625 | Caribbean | | Tibes A, Puerto Rico | AD600-900 | Mandible (R) | [4] |
| 3 | MS10626 | Caribbean | | Tibes B, Puerto Rico | AD900-1200 | Mandible (L) | [4] |
| 4 | MS10709 | Caribbean | | Finca Valencia, Puerto Rico | Post AD1000 | Mandible Fragment (R) | [7] |
| 5 | MS10710 | Caribbean | | Finca Valencia, Puerto Rico | Post AD1000 | Mandible Fragment (R) |  |
| 6 | MS10711 | Caribbean | | Finca Valencia, Puerto Rico | Post AD1000 | Mandible (R) |  |
| 7 | MS10712 | Caribbean | | Finca Valencia, Puerto Rico | Post AD1000 | Mandible (R) |  |
| 8 | MS10627 | Caribbean | | Grand Bay, Carriacou | AD985-1030 | Maxilla (R) | [10] |
| 9 | MS10630 | Caribbean | | Coconut Hall, Antigua | AD930-1190 | Mandible (L) | [16] |
| 10 | MS10631 | Caribbean | | Giraudy, St Lucia | AD1200-1400 | Atlas, Lumbar Vertebrae | [79] |
| 11 | MS10632 | Caribbean | | Coconut Walk, Nevis | AD970-1170 | Femur Epiphysis (R) | [80] |
| 12 | MS10633 | Caribbean | | Coconut Walk, Nevis | AD970-1170 | Femur Epiphysis (L) |  |
| 13 | MS10634 | Caribbean | | Coconut Walk, Nevis | AD970-1170 | Femur Epiphysis (L) |  |
| 14 | MS10636 | Colombia | | El Venado | AD800-1000 | Mandible (R) | [17] |
| 15 | MS10637 | Colombia | | El Venado | AD800-1000 | Cranial Fragments |  |
| 16 | MS10638 | Colombia | | El Venado | AD1250-1600 | Mandible (L) |  |
| 17 | MS10639 | Colombia | | El Venado | AD1250-1600 | Mandible (R) |  |
| 18 | MS10640 | Colombia | | El Venado | AD1000-1250 | Mandible (L) |  |
| 19 | MS10641 | Colombia | | El Venado | AD800-1000 | Mandible (R) |  |
| 20 | MS10672 | Colombia | | Madrid Site | 200-100BC | Mandible (R) | [81] |
| 21 | MS10673 | Colombia | | Aguazuque I | 1885-1815BC | Incisors and molar | [21] |
| 22 | MS10674 | Colombia | | Aguazuque I | 1885-1815BC | Molar |  |
| 23 | MS10675 | Colombia | | Aguazuque I | 1885-1815BC | Incisors |  |
| 24 | MS10676 | Colombia | | Checua II | 5940-5760BC | Premaxilla Fragment and Incisor | [22] |
| 25 | MS10677 | Colombia | | Checua II | 5940-5760BC | Maxilla Fragment with Molar |  |
| 26 | MS10678 | Colombia | | Checua II | 5940-5760BC | Molars |  |
| 27 | MS10679 | Colombia | | Checua II | 5940-5760BC | Maxilla and Molars |  |
| 28 | MS10680 | Colombia | | Checua II | 5940-5760BC | Incisors |  |
| 29 | MS10681 | Peru | | Kuelap | AD1100-1535 | Femur Fragment | [23] |
| 30 | MS10682 | Peru | | Kuelap | AD1100-1535 | Mandible Fragment |  |
| 31 | MS10683 | Peru | | Kuelap | AD1100-1535 | Humerus Fragment |  |
| 32 | MS10684 | Peru | | Kuelap | AD1100-1535 | Femur Fragment |  |
| 33 | MS10685 | Peru | | Kuelap | AD1100-1535 | Femur Fragment |  |
| 34 | MS10699 | | Peru | Pachacamac | AD1000-1500 | Mandible (R) | [82] |
| 35 | MS10700 | | Peru | Pachacamac | AD1500 | Mandible (L) |  |
| 36 | MS10701 | | Peru | Pachacamac | AD1500 | Mandible (R) |  |
| 37 | MS10702 | | Peru | Pachacamac | AD1500 | Mandible (R) |  |
| 38 | MS10703 | | Peru | Pachacamac | AD1000-1500 | Mandible (R) |  |
| 39 | MS10704 | | Peru | Pachacamac | AD1000-1500 | Mandible (L) |  |
| 40 | MS10705 | | Peru | Pachacamac | AD600-1000 | Mandible (L & R) |  |
| 41 | MS10706 | | Peru | Pachacamac | AD600-1000 | Mandible (R) |  |
| 42 | MS10707 | | Peru | Pachacamac | AD600-1000 | Mandible (R) |  |
| 43 | MS10628 | | Peru | Moqi | AD1400-1532 | Mandible (L) | [32] |
| 44 | MS10629 | | Peru | Moqi | AD1400-1532 | Mandible (L) |  |
| 45 | MS10645 | | Peru | Moqi | AD1400-1532 | Mandible (L) |  |
| 46 | MS10646 | | Peru | Moqi | AD1400-1532 | Mandible (L) |  |
| 47 | MS10647 | | Peru | Moqi | AD1400-1532 | Mandible (L) |  |
| 48 | MS10648 | | Peru | Moqi | AD1400-1532 | Mandible (L) |  |
| 49 | MS10649 | | Peru | Moqi | AD1400-1532 | Innominate (L) |  |
| 50 | MS10642 | | Peru | Lo Demas | AD1480-1540 | Femur (L) | [35] |
| 51 | MS10643 | | Peru | Lo Demas | AD1480-1540 | Mandible (L) |  |
| 52 | MS10644 | | Peru | Lo Demas | AD1480-1540 | Mandible (L) |  |
| 53 | MS10713 | | Peru | Lo Demas | AD1480-1540 | Rib (L) |  |
| 54 | MS10714 | | Peru | Lo Demas | AD1480-1540 | Auditory Bulla (R) |  |
| 55 | MS10715 | | Peru | Lo Demas | AD1480-1540 | Auditory Bulla (L) |  |
| 56 | MS10716 | | Peru | Lo Demas | AD1480-1540 | Premaxilla Fragment (L) |  |
| 57 | MS10650 | | Peru | Torata Alta | AD1550-1600 | Mandible (L) | [38] |
| 58 | MS10717 | | Peru | Zana/Carrizales | AD1566-1600 | Mandible Fragment (L) | [83] |
| 59 | MS10718 | | Peru | Zana/Carrizales | AD1566-1600 | Mandible Fragment and 3 teeth (L) |  |
| 60 | MS10719 | | Peru | Zana/Carrizales | AD1566-1600 | Maxilla Fragment (L) |  |
| 61 | MS10651 | | Bolivia | Kala Uyuni | 900-700BC | Mandible (R) | [46] |
| 62 | MS10652 | | Bolivia | Kala Uyuni | 900-700BC | Mandible (R) |  |
| 63 | MS10653 | | Bolivia | Chrirpa, Llusco Structure | 800-750BC | Mandible (R) | [42] |
| 64 | MS10654 | | Bolivia | Chrirpa, Llusco Structure | 800-750BC | Mandible (L) |  |
| 65 | MS10655 | | Belgium (Historic) | Mons, Brussels | AD1440-1530 | Tibia Shaft | [48] |
| 66 | MS10671 | | Charleston (Historic) | Heyward-Washington House | AD1820 | Femur Fragment | [84] |
| 67 | MS10635 | | Caribbean (Modern) | Puerto Rico | modern | Mandible (L & R) | Susan deFrance |
| 68 | MrsChloe | | NZ (Modern European) | Dunedin | modern | Tissue | [56] |

Table S2. Results of sequencing success per archaeological site.

| **Period** | **Location** | **Site** | **Samples (n)** | **Amplified libraries (n)** | **Sequences over 90% coverage (n)** | **Success Rate (%)** |
| --- | --- | --- | --- | --- | --- | --- |
| Prehistoric | Jamaica | Green Castle | 1 | 1 | 0 | 0% |
|  | Puerto Rico | Tibes (A and B) | 2 | 2 | 2 | 100% |
|  |  | Finca Valencia | 4 | 4 | 4 |  |
|  | Carriacou | Grand Bay | 1 | 1 | 1 |  |
|  | Antigua | Coconut Hall | 1 | 1 | 1 |  |
|  | St Lucia | Giraudy | 1 | 1 | 0 | 0% |
|  | Nevis | Coconut Walk | 3 | 3 | 0 | 0% |
|  | Colombia | El Venado | 6 | 6 | 6 | 100% |
|  |  | Madrid Site | 1 | 1 | 1 |  |
|  |  | Aguazuque I | 3 | 1 | 1 | 33% |
|  |  | Checua II | 5 | 5 | 4 | 80% |
|  | Peru | Lo Demas | 7 | 7 | 3 | 42% |
|  |  | Moqi | 7 | 7 | 3 | 43% |
|  |  | Torata Alta | 1 | 1 | 1 | 100% |
|  |  | Kuelap | 5 | 5 | 5 |  |
|  |  | Pachacamac | 9 | 6 | 4 | 44% |
|  |  | Zana | 3 | 2 | 0 | 0% |
|  | Bolivia | Kala Uyuni | 2 | 2 | 2 | 100% |
|  |  | Llusco | 2 | 2 | 2 |  |
| Historic | Belgium | Mons | 1 | 1 | 1 | 100% |
|  | South Carolina | Charleston | 1 | 1 | 1 |  |
| Modern | Puerto Rico | San Sebastian | 1 | 1 | 1 |  |
|  | New Zealand | Dunedin | 1 | 1 | 1 |  |
| Total (% of total) | | | 68 | 62 (91%) | 44 (65%) |  |

Table S3. All samples with >90% coverage of the mitogenome (used in phylogenetic analyses). Total coverage, average read depth and percent contamination are shown.

|  | **Sample** | **Region** | **Site** | **Date** | **Total Coverage (%)** | **Average Coverage** | **Contamination Estimate (%)*** |
| --- | --- | --- | --- | --- | --- | --- | --- |
| 1 | MS10625 | Caribbean | Tibes A, Puerto Rico | AD600-900 | 99.2 | 418.4 | 0.0001 |
| 2 | MS10626 | Caribbean | Tibes B, Puerto Rico | AD900-1200 | 92 | 4.7 | 0.0002 |
| 3 | MS10709 | Caribbean | Finca Valencia, Puerto Rico | Post AD1000 | 99.1 | 344.4 | 0.0013 |
| 4 | MS10710 | Caribbean | Finca Valencia, Puerto Rico | Post AD1000 | 99.2 | 500.5 | 0.0024 |
| 5 | MS10711 | Caribbean | Finca Valencia, Puerto Rico | Post AD1000 | 99.2 | 208.9 | 0.0015 |
| 6 | MS10712 | Caribbean | Finca Valencia, Puerto Rico | Post AD1000 | 99.2 | 97.1 | 0.0073 |
| 7 | MS10627 | Caribbean | Grand Bay, Carriacou | AD985-1030 | 99 | 26.8 | 0.0005 |
| 8 | MS10630 | Caribbean | Coconut Hall, Antigua | AD930-1190 | 99.2 | 143.3 | 0.0010 |
| 9 | MS10636 | Colombia | El Venado | AD800-1000 | 100 | 249.2 | 0.0010 |
| 10 | MS10637 | Colombia | El Venado | AD800-1000 | 99.9 | 153.5 | 0.0002 |
| 11 | MS10638 | Colombia | El Venado | AD1250-1600 | 100 | 351.5 | 0.0016 |
| 12 | MS10639 | Colombia | El Venado | AD1250-1600 | 100 | 921 | 0.0002 |
| 13 | MS10640 | Colombia | El Venado | AD1000-1250 | 99.8 | 38.6 | 0.0000 |
| 14 | MS10641 | Colombia | El Venado | AD800-1000 | 100 | 83.9 | 0.0019 |
| 15 | MS10672 | Colombia | Madrid Site | 200-100BC | 100 | 306.3 | 0.0002 |
| 16 | MS10675 | Colombia | Aguazuque I | 1885-1815BC | 100 | 217.5 | 0.0006 |
| 17 | MS10677 | Colombia | Checua II | 5940-5760BC | 100 | 1706.3 | 0.0005 |
| 18 | MS10678 | Colombia | Checua II | 5940-5760BC | 100 | 572.9 | 0.0002 |
| 19 | MS10679 | Colombia | Checua II | 5940-5760BC | 100 | 239.1 | 0.0001 |
| 20 | MS10680 | Colombia | Checua II | 5940-5760BC | 99.9 | 47.9 | 0.0195 |
| 21 | MS10681 | Peru | Kuelap | AD1100-1535 | 99.5 | 1831.9 | 0.0007 |
| 22 | MS10682 | Peru | Kuelap | AD1100-1535 | 99.4 | 2902 | 0.0006 |
| 23 | MS10683 | Peru | Kuelap | AD1100-1535 | 99.5 | 3297.4 | 0.0001 |
| 24 | MS10684 | Peru | Kuelap | AD1100-1535 | 99.4 | 2406.4 | 0.0003 |
| 25 | MS10685 | Peru | Kuelap | AD1100-1535 | 99.3 | 2961.1 | 0.0007 |
| 26 | MS10699 | Peru | Pachacamac | AD1000-1500 | 98.8 | 25.6 | 0.0002 |
| 27 | MS10700 | Peru | Pachacamac | AD1500 | 99.1 | 136.9 | 0.0011 |
| 28 | MS10704 | Peru | Pachacamac | AD1000-1500 | 99 | 27.2 | 0.0000 |
| 29 | MS10707 | Peru | Pachacamac | AD600-1000 | 99.2 | 54.4 | 0.0002 |
| 30 | MS10628 | Peru | Moqi | AD1400-1532 | 99.2 | 687.4 | 0.0003 |
| 31 | MS10645 | Peru | Moqi | AD1400-1532 | 99.2 | 398.8 | 0.0002 |
| 32 | MS10649 | Peru | Moqi | AD1400-1532 | 98.6 | 23.6 | 0.0040 |
| 33 | MS10643 | Peru | Lo Demas | AD1480-1540 | 98.9 | 37.3 | 0.0022 |
| 34 | MS10715 | Peru | Lo Demas | AD1480-1540 | 98.8 | 33.7 | 0.0063 |
| 35 | MS10716 | Peru | Lo Demas | AD1480-1540 | 95.8 | 6.5 | 0.0082 |
| 36 | MS10650 | Peru | Torata Alta | AD1550-1600 | 99.5 | 1238.6 | 0.0026 |
| 37 | MS10651 | Bolivia | Kala Uyuni | 900-700BC | 99.1 | 143.4 | 0.0010 |
| 38 | MS10652 | Bolivia | Kala Uyuni | 900-700BC | 99.1 | 442.4 | 0.0041 |
| 39 | MS10653 | Bolivia | Llusco | 800-750BC | 99.1 | 478.5 | 0.0004 |
| 40 | MS10654 | Bolivia | Llusco | 800-750BC | 98.8 | 31.5 | 0.0003 |
| 41 | MS10655 | Belgium (Historic) | Mons, Brussels | 1550-1640 | 99.5 | 3253.1 | 0.0005 |
| 42 | MS10671 | Charleston (Historic) | Heyward-Washington House | AD1820 | 99.2 | 399.8 | 0.0002 |
| 43 | MS10635 | Caribbean (Modern) | Puerto Rico | modern | 99.2 | 623.2 | 0.0001 |
| 44 | MrsChloe | NZ (Modern European) | Dunedin | modern | 100 | 734 | 0.0000 |

*Percent contamination = number of contaminating reads/total reads per sample.

Table S4. All samples with <90% coverage of the mitogenome.

|  | **Sample** | **Region** | **Site** | **Date** | **Total Coverage(%)** | **Average Coverage** |
| --- | --- | --- | --- | --- | --- | --- |
| 1 | MS10708 | Caribbean | Green Castle, Jamaica | Post AD500 | 47.1 | 0.7 |
| 2 | MS10631 | Caribbean | Giraudy, St Lucia | AD1200-1400 | 21.1 | 0.2 |
| 3 | MS10632 | Caribbean | Coconut Walk, Nevis | AD970-1170 | - | - |
| 4 | MS10633 | Caribbean | Coconut Walk, Nevis | AD970-1170 | - | - |
| 5 | MS10634 | Caribbean | Coconut Walk, Nevis | AD970-1170 | - | - |
| 6 | MS10673* | Colombia | Aguazuque I | 1885-1815BC | - | - |
| 7 | MS10674* | Colombia | Aguazuque I | 1885-1815BC | - | - |
| 8 | MS10676* | Colombia | Checua II | 5940-5760BC | - | - |
| 9 | MS10701 | Peru | Pachacamac | AD1500 | 20.5 | 0.3 |
| 10 | MS10702 | Peru | Pachacamac | AD1500 | 78.8 | 2.4 |
| 11 | MS10703* | Peru | Pachacamac | AD1000-1500 | - | - |
| 12 | MS10705* | Peru | Pachacamac | AD600-1000 | - | - |
| 13 | MS10706* | Peru | Pachacamac | AD600-1000 | - | - |
| 14 | MS10629 | Peru | Moqi | AD1400-1532 | 82.3 | 2 |
| 15 | MS10646 | Peru | Moqi | AD1400-1532 | 29.9 | 0.4 |
| 16 | MS10647 | Peru | Moqi | AD1400-1532 | 73.6 | 1.6 |
| 17 | MS10648 | Peru | Moqi | AD1400-1532 | 52.6 | 0.8 |
| 18 | MS10642 | Peru | Lo Demas | AD1480-1540 | - | - |
| 19 | MS10644 | Peru | Lo Demas | AD1480-1540 | 68.1 | 2.3 |
| 20 | MS10713 | Peru | Lo Demas | AD1480-1540 | 62.8 | 1.1 |
| 21 | MS10714 | Peru | Lo Demas | AD1480-1540 | 15.9 | 0.2 |
| 22 | MS10717* | Peru | Zana | AD1566-1600 | - | - |
| 23 | MS10718 | Peru | Zana | AD1566-1600 | 83.5 | 2.9 |
| 24 | MS10719 | Peru | Zana | AD1566-1600 | 34.2 | 0.4 |

*Not sequenced due to inadequate library prior to sequencing.

| **Table S5. Cultural affiliations by time and geographic location of sites samples with >90% coverage of the mitogenome.** | | | | | | |  |  |  |
| --- | --- | --- | --- | --- | --- | --- | --- | --- | --- |
|  |  |  |  |  |  |  |  |  |  |
| **Date** | **Temporal Affiliation** | **N** | **North America** | **Europe** | **Caribbean** | **Colombia** | **Peru** | **Bolivia** |  |
| 1820 | Early America | 1 | Heyward-Washington House, Charleston, South Carolina |  |  |  |  |  |  |
| 1550-1640 | Early Modern Europe | 1 |  | Mons, Brussels, Belgium |  |  |  |  |  |
| AD1532-1600 | Spanish colonial | 1 |  |  |  |  | Torata Alta |  |  |
| AD1500 | Late Horizon/Spanish Colonial Period | 1 |  |  |  |  | Pachacamac |  |  |
| AD1450-1540 | Late Horizon (Inca) | 6 |  |  |  |  | Lo Demas (3), Moqi (3) |  |  |
| AD1250-1600 | Late Horizon (Inca) | 2 |  |  |  | El Venado |  |  |  |
| AD1000-1500 | Late Intermediate Period/Late Horizon | 7 |  |  |  |  | Pachacamac (2), Kuelap (5) |  |  |
| AD1000-1250 | Early Muisca | 1 |  |  |  | El Venado |  |  |  |
| post AD1000 | Late Ostionoid | 4 |  |  | Finca Valencia, PR |  |  |  |  |
| AD900-1200 | Late Ostionoid | 1 |  |  | Tibes B, PR |  |  |  |  |
| AD985-1030 | Late Suazan (Ostionoid) | 1 |  |  | Grand Bay, Carriacou |  |  |  |  |
| AD930-1190 | Mamoran-Troumassoid | 1 |  |  | Coconut Hall, Antigua |  |  |  |  |
| AD800-1000 | Late Herrera Period | 3 |  |  |  | El Venado |  |  |  |
| AD600-1000 | Middle Horizon/Late Intermediate | 1 |  |  |  |  | Pachacamac |  |  |
| AD600-900 | Early Ostionoid | 1 |  |  | Tibes A, PR |  |  |  |  |
| 200-100BC | Early Herrera Period | 1 |  |  |  | Madrid site |  |  |  |
| 900-700BC | Late Formative (Late Chiripa) | 2 |  |  |  |  |  | Kala Uyuni |  |
| 800-750BC | Late Formative (Late Chiripa) | 2 |  |  |  |  |  | Llusco, Chiripa |  |
| 1185-1815BC | Early Herrera Period | 1 |  |  |  | Aguazuque I |  |  |  |
| 5940-5760BC | Precerarmic/Archaic | 4 |  |  |  | Checua II |  |  |  |
|  |  |  |  |  |  |  |  |  |  |

Table S6. List of Genbank IDs of sequences used in the Cytochrome B phylogenetic analysis.

|  | **Genbank ID** | **Length (bp)** | **Species** | **Location** |
| --- | --- | --- | --- | --- |
| 1 | NC000884.1 | 1140 | *C. porcellus* | Europe |
| 2 | AF490405.1 | 1140 | *C. porcellus* | Colombia: Tolima Market |
| 3 | AY228361.1 | 1140 | *C. porcellus* | Chile: San Pedro de Atacama, house breed |
| 4 | AY228362.1 | 1140 | *C. porcellus* | Market Breed |
| 5 | AY228363.1 | 1140 | *C. porcellus* | Peru |
| 6 | AY245094 .1 | 1140 | *C. porcellus* | Peru: City Market |
| 7 | AY245095.1 | 1140 | *C. porcellus* | Peru: City Market |
| 8 | AY245096.1 | 1140 | *C. porcellus* | Peru: City Market |
| 9 | AY245097.1 | 1140 | *C. porcellus* | Peru: Popular Market Cusco |
| 10 | AY245098.1 | 1140 | *C. porcellus* | Peru: Popular Market Puno |
| 11 | AY245099.1 | 1140 | *C. tschudii* | Peru: Popular Market Puno |
| 12 | AY247008.1 | 1140 | *C. porcellus* | Peru: Market Arequipa |
| 13 | AY382790.1 | 1140 | *C. aperea* | Bolivia |
| 14 | AY382791.1 | 1140 | *C. aperea* | Paraguay: Concepcion |
| 15 | AY382792.1 | 1140 | *C. tschudii* | Peru: Popular Market Puno |
| 16 | AY382793.1 | 1140 | *C. porcellus* | Chile: Mercado del Agro, Arica |
| 17 | DQ017037 | 1140 | *C. porcellus* | Chile: Andina Arica |
| 18 | DQ017038 | 1140 | *C. porcellus* | Chile: Pirbright |
| 19 | DQ017039 | 1140 | *C. porcellus* | Chile: Andina Arica |
| 20 | DQ017040 | 1140 | *C. porcellus* | Chile: Andina Arica |
| 21 | DQ017041 | 1140 | *C. porcellus* | Peru: Creole Junin |
| 22 | DQ017042 | 1140 | *C. porcellus* | Ecuador: Mejocuy Auqui |
| 23 | DQ017043 | 1140 | *C. porcellus* | Ecuador: Mejocuy Auqui |
| 24 | DQ017044 | 1140 | *C. porcellus* | Bolivia: Mejocuy Nativa |
| 25 | DQ017045 | 1140 | *C. porcellus* | Bolivia: Mejocuy Nativa |
| 26 | DQ017046 | 1140 | *C. porcellus* | Peru: Mejocuy Tamborada |
| 27 | DQ017047 | 1140 | *C. porcellus* | Peru: Mejocuy Tamborada |
| 28 | DQ017048 | 1140 | *C. tschudii* | Chile: Wild Lluta |
| 29 | DQ017049 | 1140 | *C. tschudii* | Chile: Wild Lluta |
| 30 | DQ017050 | 1140 | *C. tschudii* | Chile: Wild Lluta |
| 31 | DQ017051 | 1140 | *C. tschudii* | Chile: Wild Lluta |
| 32 | DQ017052 | 1140 | *C. tschudii* | Chile: Wild Lluta |
| 33 | DQ017053 | 1140 | *C. tschudii* | Peru: Wild Chiguata |
| 34 | GU067538.1 | 1140 | *C. aperea* | Bolivia: La Paz |
| 35 | GU136721.1 | 1140 | *Hydrochoerus hydrochaeris* | South America |
| 36 | GU136722.1 | 1140 | *Kerodon rupestris* | South America |
| 37 | GU136723.1 | 1140 | *Dolichotis salinicola* | South America |
| 38 | GU136724.1 | 1140 | *Dolichotis patagonum* | South America |
| 39 | GU136725.1 | 1140 | *Microcavia niata* | South America |
| 40 | GU136726.1 | 1140 | *C. tschudii sodalis* | Argentina:juyjuy |
| 41 | GU136727.1 | 1140 | *C. tschudii* | Peru:Ica |
| 42 | GU136728.1 | 1140 | *C. tschudii* | Peru:Ica |
| 43 | GU136729.1 | 1140 | *C. tschudii* | Bolivia: Santa Cruz |
| 44 | GU136730.1 | 1140 | *C. tschudii osgoodi* | Peru: Puno |
| 45 | GU136731.1 | 1140 | *C. tschudii* | Peru: Cuzco |
| 46 | GU136732.1 | 1140 | *C. porcellus* | Bolivia: La Paz |
| 47 | GU136733.1 | 1140 | *C. porcellus* | Colombia: Tolima |
| 48 | GU136734.1 | 1140 | *C. magna* | Uruguay: Rocha |
| 49 | GU136735.1 | 1140 | *C. magna* | Uruguay: Rocha |
| 50 | GU136736.1 | 1140 | *C. magna* | Uruguay: Rocha |
| 51 | GU136737.1 | 1140 | *C. fulgida* | Brazil: Mina Gerais |
| 52 | GU136740.1 | 1140 | *C. aperea hypoleuca* | Paraguay: Concepcion |
| 53 | GU136741.1 | 1140 | *C. aperea* | Bolivia: Santa Cruz |
| 54 | GU136742.1 | 1140 | *C. aperea* | Bolivia: Santa Cruz |
| 55 | GU136743.1 | 1140 | *C. aperea* | Bolivia: Beni |
| 56 | GU136744.1 | 1140 | *C. aperea pamparum* | Argentina: Buenos Aires |
| 57 | GU136745.1 | 1140 | *C. aperea pamparum* | Argentina: Buenos Aires |
| 58 | GU136746.1 | 1140 | *C. aperea pamparum* | Argentina: Santa Fe |
| 59 | GU136747.1 | 1140 | *C. aperea pamparum* | Argentina: Santa Fe |
| 60 | GU136748.1 | 1140 | *C. aperea pamparum* | Argentina: Santa Fe |
| 61 | GU136749.1 | 1140 | *C. aperea pamparum* | Argentina: Buenos Aires |
| 62 | GU136750.1 | 1140 | *C. aperea pamparum* | Argentina: Buenos Aires |
| 63 | GU136751.1 | 1140 | *C. aperea pamparum* | Uruguay: Rio Negro |
| 64 | GU136752.1 | 1140 | *C. aperea* | Bolivia: Santa Cruz |
| 65 | GU136753.1 | 1140 | *C. aperea* | Bolivia: Santa Cruz |
| 66 | GU136754.1 | 1140 | *C. aperea* | Bolivia: Santa Cruz |
| 67 | GU136755.1 | 1140 | *C. aperea guianae* | Colombia: Meta |
| 68 | GU136756.1 | 1140 | *C. aperea guianae* | Colombia: Meta |
| 69 | GU136757.1 | 1140 | *C. aperea guianae* | Colombia: Meta |
| 70 | GU136758.1 | 1140 | *C. aperea anolaimae* | Colombia: Cundinamarca |
| 71 | GU136759.1 | 1140 | *C. aperea guianae* | Suriname: Nickeri |
| 72 | GU136760.1 | 791 | *C. patzelti* | Ecuador: Chimborazo |
| 73 | GU136761.1 | 428 | *C. patzelti* | Ecuador: Chimborazo |
| 74 | HM447146.1 | 1140 | *C. porcellus* | Colombia: Pupiales |
| 75 | HM447147.1 | 1140 | *C. porcellus* | Colombia: Pupiales |
| 76 | HM447148.1 | 1140 | *C. porcellus* | Colombia: Pupiales |
| 77 | HM447149.1 | 1140 | *C. porcellus* | Colombia: Pupiales |
| 78 | HM447150.1 | 1140 | *C. porcellus* | Colombia: Potosi |
| 79 | HM447151.1 | 1140 | *C. porcellus* | Colombia: Potosi |
| 80 | HM447152.1 | 1140 | *C. porcellus* | Colombia: Potosi |
| 81 | HM447153.1 | 1140 | *C. porcellus* | Colombia: Potosi |
| 82 | HM447154.1 | 1140 | *C. porcellus* | Colombia: Potosi |
| 83 | HM447155.1 | 1140 | *C. porcellus* | Colombia: Obonuco |
| 84 | HM447156.1 | 1140 | *C. porcellus* | Colombia: Obonuco |
| 85 | HM447157.1 | 1140 | *C. porcellus* | Colombia: Obonuco |
| 86 | HM447158.1 | 1140 | *C. porcellus* | Colombia: Obonuco |
| 87 | HM447159.1 | 1140 | *C. porcellus* | Colombia: Udenar |
| 88 | HM447160.1 | 1140 | *C. porcellus* | Colombia: Udenar |
| 89 | HM447161.1 | 1140 | *C. porcellus* | Colombia: Udenar |
| 90 | HM447162.1 | 1140 | *C. porcellus* | Colombia: Udenar |
| 91 | HM447163.1 | 1140 | *C. porcellus* | Colombia: Udenar |
| 92 | HM447164.1 | 1140 | *C. porcellus* | Colombia: Udenar |
| 93 | HM447165.1 | 1140 | *C. porcellus* | Colombia: Udenar |
| 94 | HM447166.1 | 1140 | *C. porcellus* | Colombia: Udenar |
| 95 | HM447167.1 | 1140 | *C. porcellus* | Colombia: Botana |
| 96 | HM447168.1 | 1140 | *C. porcellus* | Colombia: Botana |
| 97 | HM447169.1 | 1140 | *C. porcellus* | Colombia: Botana |
| 98 | HM447170.1 | 1140 | *C. porcellus* | Colombia: Botana |
| 99 | HM447171.1 | 1140 | *C. porcellus* | Colombia: Botana |
| 100 | HM447172.1 | 1140 | *C. porcellus* | Colombia: Botana |
| 101 | HM447173.1 | 1140 | *C. porcellus* | Colombia: Botana |
| 102 | HM447174.1 | 1140 | *C. porcellus* | Colombia: Botana |
| 103 | HM447175.1 | 1140 | *C. porcellus* | Colombia: Botana |
| 104 | HM447176.1 | 1140 | *C. porcellus* | Colombia: Botana |
| 105 | HM447177.1 | 1140 | *C. porcellus* | Colombia: Botana |
| 106 | HM447178.1 | 1140 | *C. porcellus* | Colombia: Botana |
| 107 | HM447179.1 | 1140 | *C. porcellus* | Colombia: Jose M Hernandez |
| 108 | HM447180.1 | 1140 | *C. porcellus* | Colombia: Jose M Hernandez |
| 109 | HM447181.1 | 1140 | *C. porcellus* | Colombia: Jose M Hernandez |
| 110 | HM447182.1 | 1140 | *C. porcellus* | Colombia: Jose M Hernandez |
| 111 | HM447183.1 | 1140 | *C. porcellus* | Colombia: Pasto |
| 112 | HM447184.1 | 1140 | *C. porcellus* | Colombia: Pasto |
| 113 | HM447185.1 | 1140 | *C. porcellus* | Colombia: Pasto |
| 114 | HM447186.1 | 1140 | *C. porcellus* | Colombia: Pupiales |
| 115 | HM447187.1 | 1140 | *C. porcellus* | Colombia: Pupiales |
| 116 | KT439327.1 | 1140 | *C. aperea* | Brazil |

**Supplementary Information References**

1. Curet LA, Stringer LM. 2010 *Tibes: People, Power, and Ritual at the Center of the Cosmos*. Tuscaloosa: University of Alabama Press.

2. Antonio Curet L, Newsom LA, deFrance SD. 2006 Prehispanic social and cultural changes at Tibes, Puerto Rico. *J. F. Archaeol.* **31**, 23–39.

3. deFrance S. 2010 Chiefly fare or who’s feeding the cacique? Equality in animal use at the Tibes ceremonial center, Puerto Rico. In *Anthropological Approaches to Zooarchaeology: Colonialism, Complexity and Animal Transformations* (eds D Campana, P Crabtree, S DeFrance, J Lev-Tov, AM Choyke), pp. 76–89. Oxford: Oxbow Books.

4. deFrance S, Hadden CS, LeFebvre MJ, DuChemin GR. 2010 Faunal use at the Tibes ceremonial site. In *Tibes: people, power, and ritual at the center of the cosmos* (eds LA Curet, LM Stringer), Tuscaloosa: University of Alabama Press.

5. Solís Magaña C, Rodríguez M. 2000 Final Phase II Archaeological Evaluation for the Finca Valencia Site North Coast Superaqueduct Project Municipality of Arecibo, Puerto Rico. *Prep. by LAW Environ. Thames-Dick Superaqueduct Partners, Guaynabo, Puerto Rico*

6. LeFebvre MJ, deFrance SD. 2014 Guinea Pigs in the Pre-Columbian West Indies. *J. Isl. Coast. Archaeol.* **9**, 16–44. (doi:10.1080/15564894.2013.861545)

7. Wing E. 2000 Guinea pig ( Cavia porcellus) remains from Finca Valencia (NCS-1), Northwest Puerto Rico, contribution to Phase II Zooarchaeology at the Finca Valencia (NCS-1) and NCS-4 Site, Northwest Puerto Rico. *Final Phase II Archaeol. Eval. Finca Val. Site North Coast Superaqueduct Proj. Munic. Arecibo, Puerto Rico.*

8. Reimer PJ. 2013 IntCal and marine 13 radiocarbon age calibration curves 0-50,000 year cal BP. *Radiocarbon* **55**, 1869–1887.

9. Fitzpatrick SM *et al.* 2009 Precolumbian settlements on Carriacou, West Indies. *J. F. Archaeol.* **34**, 247–266.

10. LeFebvre MJ. 2007 Zooarchaeological analysis of prehistoric vertebrate exploitation at the Grand Bay Site, Carriacou, West Indies. *Coral Reefs* **26**, 931–944.

11. Fitzpatrick SM, Kaye Q, Feathers J, Pavia JA, Marsaglia KM. 2009 Evidence for inter-island transport of heirlooms: luminescence dating and petrographic analysis of ceramic inhaling bowls from Carriacou, West Indies. *J. Archaeol. Sci.* **36**, 596–606. (doi:10.1016/j.jas.2008.08.007)

12. Giovas CM, LeFebvre MJ, Fitzpatrick SM. 2012 New records for prehistoric introduction of Neotropical mammals to the West Indies: evidence from Carriacou, Lesser Antilles. *J. Biogeogr.* **39**, 476–487. (doi:10.1111/j.1365-2699.2011.02630.x)

13. Giovas CM. 2018 Continental connections and insular distributions: deer bone artifacts of the PreColumbian West Indies—a review and synthesis with new records. *Lat. Am. Antiq.* **29**, 27–43.

14. Fuess MT. 1995 Preliminary archaeological research of prehistoric Amerindian sites on Antigua, Northern Lesser Antilles. In *Proceedings of the XV International Congress for Caribbean Archaeology* (eds RE Alegria, M Rodrigues), pp. 173–180. San Juan: University of Turabo.

15. Rouse I. 1992 *The Tainos: rise and decline of the people who greeted Columbus*. Yale University Press.

16. Healy P, Thornton E, Fuess M. 2003 The Post Saladoid Faunal Assemblage from The Coconut Hall Site, Antigua, West Indies. In *In Proceedings from the XX International Congress for Caribbean Archaeology. Museo del Hombre Dominicano and Fundación García Arévalo, Santo Domingo* (eds C Tavárez, MA García Arévalo), pp. 189–200. San Juan: University of Turabo.

17. Boada Rivas AM. 2007 The Evolution of Social Hierarchy in a Muisca Chiefdom of the Northern Andes of Colombia. In *University of Pittsburgh Memoirs in Latin American Archaeolgoy*,

18. Rodríguez JV, Cifuentes A. 2005 Un yacimiento formativo ritual en el entorno de la antigua laguna de La Herrera, Madrid, Cundinamarca. *Maguaré* , 102–131.

19. Rodríguez JV. 2011 *Los Chibchas: hijos del sol, la luna y los Andes. Orígenes de su diversidad*. Bogotá: Universidad Nacional de Colombia, Sede Bogotá, Facultad de Ciencias Humanas, Departamento de Antropología.

20. Correal Urrego G. 1990 Aguazuque: evidencias de cazadores, recolectores y plantadores en la altiplanicie de la Cordillera Oriental. *Fund. Investig. Arqueol. Nac. Publicaciones*.

21. Martínez-Polanco MF. 2016 El Cuy (Cavia Sp.), Un Recurso Alimenticio Clave en Aguazuque, Un Sitio Arqueológico de la Sabana de Bogotá, Colombia. *Lat. Am. Antiq.* **27**, 512–526. (doi:10.7183/1045-6635.27.4.512)

22. Groot AM. 1992 *Checua: una secuencia cultural entre 8500 y 3000 años antes del presente*. Fundación de Investigaciones Arqueológicas Nacionales (Banco de la República).

23. Narváez Vargas A. 1996 La fortaleza de Kuelap. *Arkinka* **13**, 90–98.

24. Narváez Vargas A. 2013 Kuelap: Centro del poder politico y religioso de los chachapoyas. In *Los Chachapoyas Colección Arte y Tesoros del Perú* (ed F Kauffmann-Doig), pp. 87–160. Lima: Banco de Crédito del Perú.

25. Church WB, von Hagen A. 2008 Chachapoyas: Cultural development at an Andean cloud forest crossroads. In *Handbook of South American Archaeology* (eds H Silverman, WH Isbell), pp. 903–926. New York: Springer.

26. Guengerich A. 2015 Settlement organization and architecture in Late Intermediate Period Chachapoyas, Northeastern Peru. *Lat. Am. Antiq.* **26**, 362–381.

27. Eeckhout P. 2013 Change and permanency on the coast of ancient Peru: the religious site of Pachacamac. *World Archaeol.* **45**, 119–142.

28. Eeckhout P. 2003 Ancient monuments and patterns of power at Pachacamac, central coast of Peru. *BAVA* **23**, 139–182.

29. Owens LS, Eeckhout P. 2015 To the god of death, disease, and healing. Social bioarchaeology of cemetery I at Pachacamac. In *Funerary Practices and Models in the Ancient Andes. The Return of the Living Dead* (eds P Eeckhout, LS Owens), pp. 158–185. New York: Cambridge University Press.

30. Eeckhout P. 2017 Ofrendas, rituales, peregrinaciones y ancestros en la arqueología de Pachacamac. In *Pachacamac el Oráculo en el Horizonte Marino del Sol Poniente* (ed D Pozzi-Escot), pp. 222–237. Lima: Banco de Crédito del Perú.

31. Erauw C, Pigière F, Eeckhout P. 2017 El sitio sagrado de Pachacamac: Nuevos enfoques sobre las ofrendas de animales. In *III Reunión Académica del Grupo de Zooarqueología Neotropical*, San José.

32. Zori C, Dahlstedt A, Carty N, Gordillo Begazo J, Knudson K. 2017 Local people performing empire: Architecture, mortuary practices and geographic origins of the inhabitants of Moqi, Upper Locumba Valley, Peru. In *Society for American Archaeology 82nd Annual Meeting*, Vancouver.

33. Sandweiss DH. 1992 The Archaeology of Chincha Fishermen: Specialization and Status in Inka Peru. *Carnegie Museum Nat. Hist. Bull. 29*

34. Rostworowski de Diez Canseco M. 1970 Mercaderes del Valle de Chincha en la Época Prehispanica: Un Documento y unos Comentarios. *Rev. Española Antropol. Am.* **5**, 135–177.

35. Sandweiss DH, Wing ES. 1997 Ritual Rodents: The Guinea Pigs of Chincha, Peru. *J. F. Archaeol.* **24**, 47. (doi:10.2307/530560)

36. Rice PM. 2012 Torata Alta: An Inka administrative center and Spanish colonial reducción in Moquegua, Peru. *Lat. Am. Antiq.* **23**, 3–28.

37. Van Buren M. 1993 Community and Empire in Southern Peru: The Site of Torata Alta under Spanish Rule. University of Arizona, Tucson.

38. deFrance SD. 1993 Ecological imperialism in the South-Central Andes: Faunal data from Spanish colonial settlements in the Moquegua and Torata valleys. University of Florida Press.

39. Hastorf CA. 2003 Community with the ancestors: ceremonies and social memory in the Middle Formative at Chiripa, Bolivia. *J. Anthropol. Archaeol.* **22**, 305–332.

40. Bruno MC, Whitehead WT. 2003 Chenopodium cultivation and Formative Period agriculture at Chiripa, Bolivia. *Lat. Am. Antiq.* **14**, 339–355.

41. Bruno MC. 2014 Beyond raised fields: Exploring farming practices and processes of agricultural change in the ancient Lake Titicaca Basin of the Andes. *Am. Anthropol.* **116**, 130–145.

42. Moore KM, Steadman D, deFrance SD. 1999 Herds, fish, and fowl in the domestic and ritual economy of Formative Chiripa. In *Early Settlement in Chiripa, Bolivia: Research of the Taraco Archaeol Project* (ed CA Hastorf), pp. 105–116. Berkeley: Archaeological Research Facility, University of California at Berkelely.

43. Whitehead WT. 1999 Radiocarbon dating. In *Radiocarbon dating, Early Settlement in Chiripa, Bolivia: Research of the Taraco Archaeol Project No. 57* (ed CA Hastorf), pp. 17–22. Berkeley: Archaeological Research Facility, University of California at Berkelely.

44. Bandy MS, Hastorf CA. 2007 Kala Uyuni: an early political center in the southern Lake Titicaca basin: 2003 Excavations of the Taraco Archaeol Project. In *Contributions of the University of Calfilornia Archaeollogical Research Facility, University of Calfornia at Berkeley*, p. 64.

45. Roddick AP, Bruno MC, Hastorf CA. 2014 Political centers in context: depositional histories at Formative Period Kala Uyuni, Bolivia. *J. Anthropol. Archaeol.* **36**.

46. Moore KM, Bruno M, Capriles JM, Hastorf CA. 2010 Integrated contextual approaches to understanding past activities using plant and animal remains from Kala Uyuni, Lake Titicaca, Bolivia. In *Integrating Zooarchaeology and Paleoethnobotany: A Consideration of Issues, Methods, and Cases* (eds AM VanDerwarker, TM Peres), pp. 173–203. New York: Springer.

47. Ansieau C, Denis M. 2009 Fouilles preventives d’une parcelle sise a Mons, rue Jean Lescarts (Ht.). *Archaeol. Mediaev.* **32**, 81–84.

48. Pigière F, Van Neer W, Ansieau C, Denis M. 2012 New archaeozoological evidence for the introduction of the guinea pig to Europe. *J. Archaeol. Sci.* **39**, 1020–1024.

49. Piérard C. 2006 Mons au fil des siècles, La ville du VIIe siècle à 1861. In *Images de Mons en Hainaut du XVIe au XIXe siècle* (ed M-T Isaac), pp. 121–132. Bruxelles: La Renaissance du livre.

50. Zierden MA, Reitz EJ, Pavao-Zuckerman B, Reitsema LJ, Manzano BL. 2018 What is this bird? The quest to identify parrot remains from the Heyward-Washington House, Charleston, South Carolina. *Southeast. Archaeol.* , 1–11. (doi:10.1080/0734578X.2018.1555407)

51. Knapp M, Clarke AC, Horsburgh KA, Matisoo-Smith EA. 2012 Setting the stage–building and working in an ancient DNA laboratory. *Ann. Anatomy-Anatomischer Anzeiger* **194**, 3–6.

52. Rohland N, Hofreiter M. 2007 Ancient DNA extraction from bones and teeth. *Nat Protoc* **2**, 1756–1762. (doi:10.1038/nprot.2007.247)

53. Matisoo-Smith EA *et al.* 2016 A European Mitochondrial Haplotype Identified in Ancient Phoenician Remains from Carthage, North Africa. *PLoS One* **11**, e0155046.

54. Maricic T, Whitten M, Pääbo S. 2010 Multiplexed DNA sequence capture of mitochondrial genomes using PCR products. *PLoS One* **5**, e14004.

55. Greig K, Boocock J, Prost S, Horsburgh KA, Jacomb C, Walter R, Matisoo-Smith E. 2015 Complete mitochondrial genomes of New Zealand’s first dogs. *PLoS One* **10**, e0138536.

56. Lord E, Collins C, deFrance SD, LeFebvre MJ, Matisoo-Smith E. 2018 Complete mitogenomes of ancient Caribbean Guinea pigs (Cavia porcellus). *J. Archaeol. Sci. Reports* **17**, 678–688.

57. Schubert M, Lindgreen S, Orlando L. 2016 AdapterRemoval v2: rapid adapter trimming, identification, and read merging. *BMC Res. Notes* **9**, 88. (doi:10.1186/s13104-016-1900-2)

58. Li H, Durbin R. 2009 Fast and accurate short read alignment with Burrows–Wheeler transform. *Bioinformatics* **25**, 1754–1760.

59. In press. http://broadinstitute.github.io/picard.

60. Jónsson H, Ginolhac A, Schubert M, Johnson PLF, Orlando L. 2013 mapDamage2. 0: fast approximate Bayesian estimates of ancient DNA damage parameters. *Bioinformatics* , btt193.

61. McKenna A *et al.* 2010 The Genome Analysis Toolkit: a MapReduce framework for analyzing next-generation DNA sequencing data. *Genome Res.* **20**, 1297–303. (doi:10.1101/gr.107524.110)

62. Li H *et al.* 2009 The Sequence alignment/map (SAM) format and SAMtools. *Bioinformatics* **25**, 2078–2079.

63. Wickham H. 2016 *ggplot2: elegant graphics for data analysis*. Springer.

64. Team RC. 2000 R language definition. *Vienna, Austria R Found. Stat. Comput.*

65. Kearse M *et al.* 2012 Geneious Basic: an integrated and extendable desktop software platform for the organization and analysis of sequence data. *Bioinformatics* **28**, 1647–1649.

66. Leigh JW, Bryant D. 2015 popart: full‐feature software for haplotype network construction. *Methods Ecol. Evol.* **6**, 1110–1116.

67. Burgos-Paz W, Ceron-Munoz M, Solarte-Portilla C. 2011 Genetic diversity and population structure of the Guinea pig (Cavia porcellus, Rodentia, Caviidae) in Colombia. *Genet Mol Biol* **34**, 711–718. (doi:10.1590/S1415-47572011005000057)

68. Dunnum JL, Salazar-Bravo J. 2010 Molecular systematics, taxonomy and biogeography of the genus Cavia (Rodentia: Caviidae). *J. Zool. Syst. Evol. Res.* **48**, 376–388. (doi:10.1111/j.1439-0469.2009.00561.x)

69. D’Erchia AM, Gissi C, Pesole G, Saccone C, Arnason U. 1996 The guinea-pig is not a rodent. *Nature* **381**, 597.

70. Spotorno AE, Manriquez G, Fernandez A, Marin JC, Gonzalez F, Wheeler J. 2007 Domestication of Guinea Pigs from a Southern Peru-northern Chile Wild Species and their Middle Pre-Columbian Mummies. *Quintessential Nat. Honor. Life Leg. Oliver P. Pearson* **134**, 367.

71. Spotorno AE, Valladares JP, Marin JC, Zeballos H. 2004 Molecular diversity among domestic guinea-pigs (Cavia porcellus) and their close phylogenetic relationship with the Andean wild species Cavia tschudii. *Rev. Chil. Hist. Nat.* **77**, 243–250.

72. Spotorno AE, Marin JC, Manriquez G, Valladares JP, Rico E, Rivas C. 2006 Ancient and modern steps during the domestication of guinea pigs (Cavia porcellus L.). *J. Zool.* **270**, 57–62. (doi:10.1111/j.1469-7998.2006.00117.x)

73. Drummond AJ, Rambaut A. 2007 BEAST: Bayesian evolutionary analysis by sampling trees. *BMC Evol. Biol.* **7**, 214.

74. Nguyen L-T, Schmidt HA, von Haeseler A, Minh BQ. 2015 IQ-TREE: a fast and effective stochastic algorithm for estimating maximum-likelihood phylogenies. *Mol. Biol. Evol.* **32**, 268–274.

75. Darriba D, Taboada GL, Doallo R, Posada D. 2012 jModelTest 2: more models, new heuristics and parallel computing. *Nat. Methods* **9**, 772.

76. Rambaut A, Drummond AJ. 2013 TreeAnnotator v1. 7.0. *Available as part BEAST Packag. http//beast.bio.ed.ac.uk*

77. Rambaut A. 2007 FigTree, a graphical viewer of phylogenetic trees. *See http//tree.bio.ed.ac.uk/software/figtree*

78. Allsworth-Jones P, Wesler K. 2001 Excavations at Green Castle, Jamaica, 1999-2001. In *Proceedings of the XIXth International Congress for Caribbean Archaeology*, pp. 186–193.

79. Phulgence W. 2007 Zooarchaeological Report on Pit 8 Level 1, Giraudy, St. Lucia. *Manuscr. file with Florida Museum Nat. Hist. Gainesv.*

80. Kaye Q, Fitzpatrick S, Kappers M, Thompson V. 2010 Beyond Time Team: Archaeological Investigations at Coconut Walk, Nevis, West Indies, 1st July–4th August 2010. *Pap. from Inst. Archaeol.* **20**.

81. Delgado ME. 2015 Mid and late Holocene populations changes at the Sabana de Bogotá (Northern South America) inferred from skeletal morphology and radiocarbon chronology. *Quat. Int.* **256**, 2–11.

82. Erauw C. In press. Informe arqueozoológico de la temporada 2014 de investigaciones arqueológicas en el sitio arqueológico de Pachacamac, Proyecto Ychsma.

83. Kennedy SA, VanValkenburgh P. 2016 Zooarchaeology and Changing Food Practices at Carrizales, Peru Following the Spanish Invasion. *Int. J. Hist. Archaeol.* **20**, 73–104. (doi:10.1007/s10761-015-0319-0)

84. Zierden MA, Reitz EJ. 2016 *Charleston: An Archaeology of Life in a Coastal Community*. Gainsville: University of Florida Press.
